# Development of a biomarker signature using grating-coupled fluorescence plasmonic microarray for diagnosis of MIS-C

**DOI:** 10.3389/fbioe.2023.1066391

**Published:** 2023-03-31

**Authors:** Michele Maltz-Matyschsyk, Clare K. Melchiorre, Katherine W. Herbst, Alexander H. Hogan, Kristina Dibble, Brandon O’Sullivan, Joerg Graf, Aishwarya Jadhav, David A. Lawrence, William T. Lee, Kyle J. Carson, Justin D. Radolf, Juan C. Salazar, Michael A. Lynes, Connecticut Children’s COVID Collaborative

**Affiliations:** ^1^ Department of Molecular and Cell Biology, University of Connecticut, Storrs, CT, United States; ^2^ Connecticut Children’s Medical Center, Hartford, CT, United States; ^3^ University of Connecticut Health Center, Farmington, CT, United States; ^4^ Wadsworth Center, New York State Department of Health, Albany, NY, United States; ^5^ University at Albany School of Public Health, Rensselaer, NY, United States

**Keywords:** COVID-19, MIS-C, diagnostic, biomarkers, microarray

## Abstract

Multisystem inflammatory syndrome in children (MIS-C) is a rare but serious condition that can develop 4–6 weeks after a school age child becomes infected by SARS-CoV-2. To date, in the United States more than 8,862 cases of MIS-C have been identified and 72 deaths have occurred. This syndrome typically affects children between the ages of 5–13; 57% are Hispanic/Latino/Black/non-Hispanic, 61% of patients are males and 100% have either tested positive for SARS-CoV-2 or had direct contact with someone with COVID-19. Unfortunately, diagnosis of MIS-C is difficult, and delayed diagnosis can lead to cardiogenic shock, intensive care admission, and prolonged hospitalization. There is no validated biomarker for the rapid diagnosis of MIS-C. In this study, we used Grating-coupled Fluorescence Plasmonic (GCFP) microarray technology to develop biomarker signatures in pediatric salvia and serum samples from patients with MIS-C in the United States and Colombia. GCFP measures antibody-antigen interactions at individual regions of interest (ROIs) on a gold-coated diffraction grating sensor chip in a sandwich immunoassay to generate a fluorescent signal based on analyte presence within a sample. Using a microarray printer, we designed a first-generation biosensor chip with the capability of capturing 33 different analytes from 80 μL of sample (saliva or serum). Here, we show potential biomarker signatures in both saliva and serum samples in six patient cohorts. In saliva samples, we noted occasional analyte outliers on the chip within individual samples and were able to compare those samples to 16S RNA microbiome data. These comparisons indicate differences in relative abundance of oral pathogens within those patients. Microsphere Immunoassay (MIA) of immunoglobulin isotypes was also performed on serum samples and revealed MIS-C patients had several COVID antigen-specific immunoglobulins that were significantly higher than other cohorts, thus identifying potential new targets for the second-generation biosensor chip. MIA also identified additional biomarkers for our second-generation chip, verified biomarker signatures generated on the first-generation chip, and aided in second-generation chip optimization. Interestingly, MIS-C samples from the United States had a more diverse and robust signature than the Colombian samples, which was also illustrated in the MIA cytokine data. These observations identify new MIS-C biomarkers and biomarker signatures for each of the cohorts. Ultimately, these tools may represent a potential diagnostic tool for use in the rapid identification of MIS-C.

## Introduction

Acute pediatric SARS-CoV-2 infections, although usually mild, have hospitalized over 150,000 children since the coronavirus disease-2019 (COVID-19) pandemic began (https://covid.cdc.gov/covid-data-tracker/#new-hospital-admissions). In rare instances, two to 6 weeks after an acute infection, children can develop a severe inflammatory disorder known as multisystem inflammatory syndrome in children (MIS-C) (Guimarães et al., 2021; Radia et al., 2021). The syndrome is non-specific and is associated with, but not limited to, the following symptoms: abdominal pain, diarrhea, vomiting, rashes, red eyes, red or swollen hands/feet, red cracked lips, cough, sore throat, fever, cardiovascular dysfunction, and respiratory dysfunction (Feldstein et al., 2020; Ramaswamy et al., 2020; Guimarães et al., 2021; Radia et al., 2021; Algarni and Alghamdi. 2022). According to the CDC as of 30 January 2023 in the US there have been 9,344 MIS-C patients (“CDC COVID Data Tracker: Multisystem Inflammatory Syndrome in Children (MIS-C)” n.d.). Half of children with MIS-C are admitted to the intensive care unit (ICU) and 76 children have died from MIS-C. Unfortunately, MIS-C symptoms and the associated immune response mimics other inflammatory diseases. For example, Kawasaki disease (an acute and self-limited vasculitis of unknown etiology) shares many clinical and laboratory markers with MIS-C (e.g., fever, rash, red eyes, inflammatory markers) leading to misdiagnosis and delaying definitive management (Newburger et al., 2016; Phamduy et al., 2021).

**FIGURE 1:**
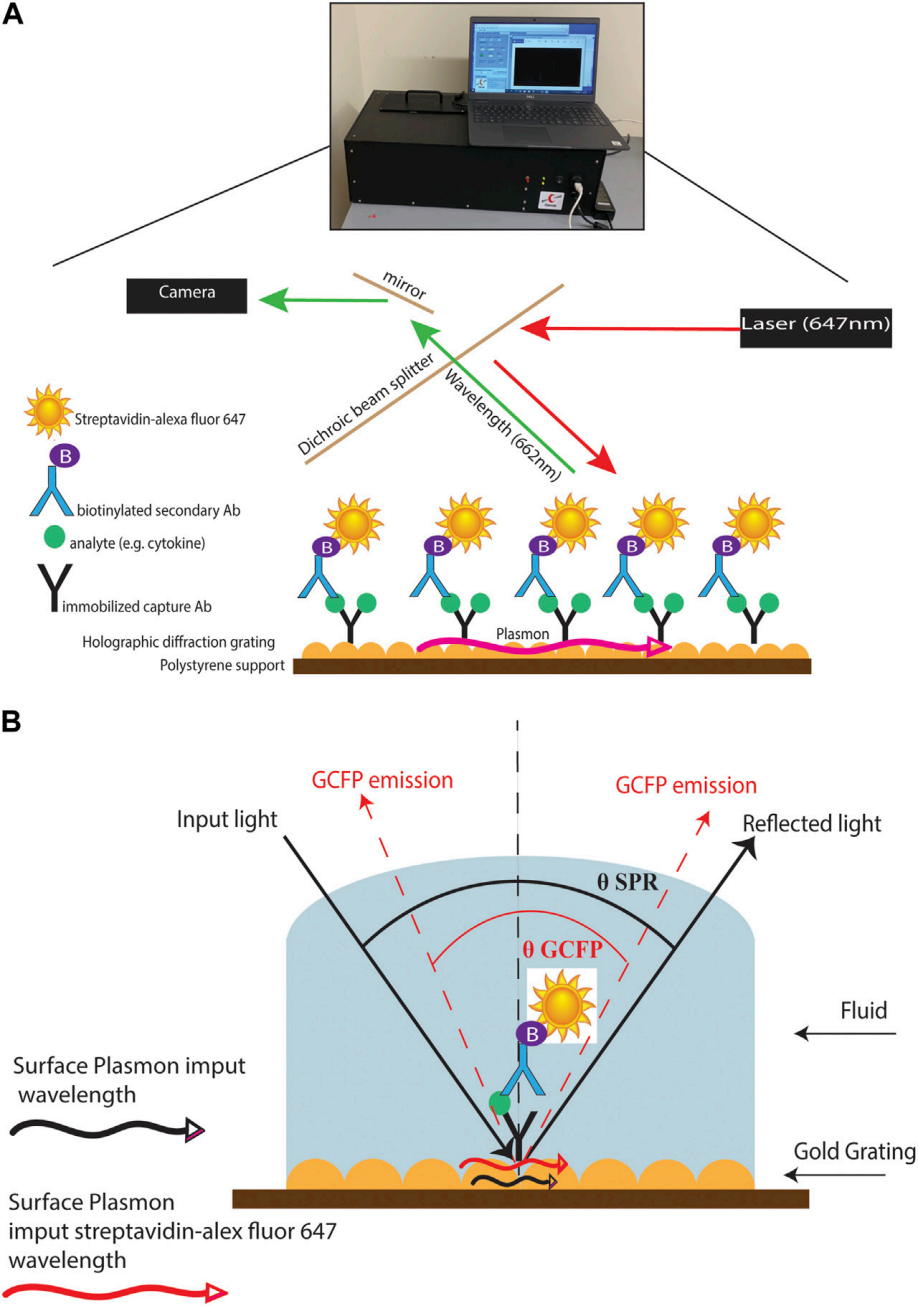
GCFP assay and wavelength pathway. (**A)** Capture antibodies are immobilized onto a gold-coated nanoscale grating surface chip by pin spotting. Chips were then fitted with plexiglass windows and gaskets to create a flow cell for fluid to pass over the surface of the printed chip. Fluid containing potential analytes can then be run over the assembled chip, followed by biotinylated secondary antibodies and then streptavidin-Alexafluor647, with washes in between steps with PSB-T. The chip can then be placed in a GCFP reader where a laser illuminates the chip and the fluorescence intensity is collected with a camera and each ROI is analyzed using Enhanced Fluorescence Reader software, V2.3. **(B)**. Under normal SPR optical conditions, the energy of the light excites electron density oscillation (the plasmon) within the metal coating on the sensor chip, reducing the intensity of the reflected light (Black lines). Using the diffraction grating on the chip, the wave vector of the illuminating beam of light can be matched with the plasmon wave vector (red lines). The gold grating plus the use of a fluorophore increases the collected light, thereby enhancing the signal (red lines).

Diagnosing MIS-C and Kawasaki (and atypical Kawasaski Disaease) often depends on blood laboratory markers; however, venipunctures can be invasive and traumatic, and it can be difficult to collect substantial blood volumes in pediatric patients (Bagnasco et al., 2012). The development of a simple and fast diagnostic tool that requires small volumes of serum or saliva to distinguish between these inflammatory diseases would help decrease diagnostic time and allow for optimized treatment options. (Li et al., 2020; Mahmoud et al., 2021). Current approaches to the analysis of biofluids using bead-based multiplex ELISA immunoassays, multi-parameter flow cytometry, reverse phase arrays, 2-D gel electrophoresis, 2-D differential in-gel electrophoresis (2-D DIGE), and antibody (Ab) microarrays can be time consuming, demanding of larger sample sizes, and labor intensive. (Amanullah et al., 2002; Noordin and Nurulhasanah, 2013). In this study, we created a novel grating-coupled fluorescent plasmonics (GCFP) microarray assay using 33 analytes to define the biosignatures of children effected by SARS-CoV-2 infection. In previous studies, GCFP has been demonstrated to be a highly sensitive tool for exploring proteomic profiles in both serum and saliva (Molony et al., 2012; Rice et al., 2012; Chou et al., 2020; Cady et al., 2021). In brief, this technology is on based surface plasmon resonance (SPR): the physical phenomenon of energy transfer at a metal-dielectric interface (Figures 1A,B) (Unfricht et al., 2005; Jin et al., 2006; Marusov et al., 2012; Molony et al., 2012; Chou et al., 2020). Under specific optical conditions, the energy of the light excites electron density oscillation (the plasmon) within the metal coating on the sensor chip, reducing the intensity of the reflected light. Using the diffraction grating on the chip, the wave vector of the illuminating beam of light can be matched with the plasmon wave vector. The gold grating plus the use of a fluorophore increases the collected light, thereby enhancing the signal (Figure 1B) (Reilly et al., 2006). Here we describe a new approach to MIS-C diagnostics that can be applied to both serum and saliva analysis. We demonstrate that the modification of a gold-coated nanoscale grating surface chip with capture antibodies targeting different analytes in a biofluid sample, combined with the use of surface plasmon resonance-enhanced fluorescence, assesses biomarkers simultaneously creating a biomarker signature (including both positive and negative detection), which can potentially distinguish MIS-C from other diseases. This assay combines the detection of several biomarkers into a biomarker signature, and we hypothesize that testing numerous biomarkers increases the likelihood of developing a robust biosignature for MIS-C. We also identify potential new markers of MIS-C and optimize sensitivity of current analytes within the microarray, which along with ongoing studies will inform the design of a second-generation chip that is more specific to MIS-C.

## Materials and methods

### Biological samples

Our prospective observational study included 260 subjects, birth to ≤21 years of age, enrolled 1 April 2020, through 1 May 2022, at sites in Connecticut, United States and Cali, Colombia. After obtaining IRB approval (#21-004), subjects were enrolled in two cohorts: an experimental cohort (Cohort A) and a reference cohort (Cohort B). The experimental cohort was comprised of subjects who were positive for SARS-CoV-2 infection per antigen or PCR testing and hospitalized for COVID-19 symptoms (subgroup A1); hospitalized and meeting the Center for Disease Control’s criteria for MIS-C (subgroup A2); and non-hospitalized subjects testing positive for SARS-CoV-2 by antigen or PCR, (subgroup A3). The reference cohort was comprised of SARS-CoV-2 negative subjects who were: hospitalized with a diagnosis of Kawasaki disease (subgroup B1); hospitalized because of an acute viral infection (subgroup B2); or healthy controls undergoing routine ambulatory surgery (subgroup B3). To ensure correct subgroup assignment, cases were independently reviewed and adjudicated by three pediatric specialists. Demographic data, health history, current symptoms, and in-patient treatments and diagnostic testing results were collected at baseline. Subjects will be followed for up to 4 years *via* survey for health and SARS-CoV-2 vaccine status.

### Patient saliva processing

Patient saliva samples were stored at −80°C for at least 24 h prior to processing. The samples were placed in the biosafety cabinet and allowed to thaw at room temperature for approximately 20 min. Once thawed, the tubes were cleaned with alcohol wipes and centrifuged at 200 *g* for 15 min at 4°C. For samples to be analyzed with the grating-coupled fluorescent plasmonic biosensor chip, 500 μL of supernatant was aliquoted and 35 μL of protease inhibitor dissolved in 1 mL PBS was added (Pierce™ protease inhibitor tablet, Thermo Fisher Scientific, MA, USA) was added. For samples intended to be used in 16S RNA PacBio sequencing, 250 μL of supernatant was aliquoted and heat-inactivated for 30 min at 56°C. Once processed, samples were stored at −80°C until use.

### Patient serum processing

Sera were isolated from patient blood samples by resting the blood for approximately 30 min after collection to allow for coagulation. The non-coagulated fraction was transferred into a 15 mL tube and centrifuged at 1000 *g* for 15 min. Supernatant was then transferred to a new tube and centrifuged at 1000 *g* for 5 min. Supernatants were aliquoted into 200 μL cryovials and stored at −80°C until transferred to participating laboratories.

### Printing of grating-coupled fluorescence plasmonic (GCFP) biosensor chip

Biosensor chips were fabricated and processed as described previously with the following modifications (Molony et al., 2012; Rice et al., 2012; Chou et al., 2020; Cady et al., 2021). Capture antibodies were diluted to 250 μg/mL with PBS (Table 1) and printed on the GCFP chips using a 0.35 mm diameter microarray pin and a SpotBot II microarray printer (ArrayIt, CA, United States) or an XactII microarray printer (LabNEXT, NJ, United States). Each capture antibody was spotted to create five individual regions of interest (ROI) per analyte on each biosensor chip. During printing, microarrays were kept at a relative humidity of 70% and at ambient temperature (AT) (−25°C). After printing, chips were allowed to dry at a relative humidity of 70% and AT (−25°C) for 1 h. Chips were then transferred to a 50 mL polypropylene tube containing desiccant and stored at AT for up to 4 weeks, with no significant signal loss (data not shown).

**TABLE 1:** Regions of interest used on first generation chip.

Marker class	Analytes
**Cytokines**	IL-7, IL-21, IFNg, IL-15, IL-6, IL-10, IL-17, IL-2, IL-4, IL-1b, TNFa, IL-33, IL-18 (total), IL-18BPa
**Chemokines**	CCL20, CXCL11, CCL24, CCL2, CCL3, CCL7, CXCL10, CXCL9, CCL5, CCL17, CXCL8, CX3CL1
**Interleukin receptor**	sCD25
**Kidney function**	Cystatin C
**Indicator of Inflammation**	CRP
**Range of biological processes**	Galectin-3
**Infection indicator**	Procalcitonin
**Type 1 membrane protein**	B7-1/CD80
**Protein found on antigen presenting cells**	Perforin
**Peptide involved in central and peripheral nervous systems**	Neuropeptide Y
**Serine protease that is expressed strongly in the pancreas**	Marapsin/Pancreasin
**Cell death**	LDH(A)
**Protein fragments produced when a blood clot**	D-Dimer
**Tissue damage**	Cardiotrophin-1
**Spike protein receptor**	ACE-2
**Heat shock protein**	HSP70
**Iron levels**	Ferritin

### Grating-coupled fluorescence plasmonic detection assay

Biosensor chips were processed as previously described with the following modifications (Molony et al., 2012; Rice et al., 2012; Chou et al., 2020) (Figure 1). Chips were fitted with plexiglass windows and gaskets to allow fluid to pass over the surface of the chip. The chip was then blocked with 80 μL of Superblock™ T20 (PBS) blocking buffer (Thermo Fisher Scientific, MA, United States) in a static incubation for 1 h. Each chip was then washed with PBS-T at a flow rate of 0.5 mL/min for 3 min using a peristaltic pump. PBS doped with recombinant protein (20 ng/mL) or patient samples (80 μL of serum or saliva samples diluted with PBS to 1.5 mL) were then recirculated over the chip for 90 min at 0.5 mL/min for 3 min. Each chip was then washed with PBS-T at 0.5 mL/min for 3 min. Biotinylated secondary antibody mixtures were created with all detection antibodies at a concentration of 200 ng/mL and then recirculated over the chip for 90 min. The chip was then washed with PBS-T at 0.5 mL/min for 3 min and then PBS containing 500 ng/mL of streptavidin-AlexaFluor 647 (Thermo Fisher) was recirculated over the chip for 1 h. The chip was then washed for a final time with PBS-T at 0.5 mL/min for 3 min and 70 μL of PBS was injected into the flow cell. ROIs were then identified using Ciencia software, Enhanced fluorescence Reader V2.3, and fluorescence intensity for each ROI was collected using a GCFP reader (Ciencia, Inc.). A GCFP detection ratio was used to normalize each ROI value as previously described (Cady et al., 2021)

### 16S-23S rRNA microbiome sequencing

DNA was extracted using the Complete Lyse & Purify kit (Shoreline Biome, Farmington, CT, United States). Saliva samples were thawed on ice, and between 50 μL and 200 µL of saliva was pelleted by centrifuged at 5,500 rpm for 10 min. Pellets were resuspended in nuclease-free water, then purified according to the manufacturer’s protocol. Extracted DNA was amplified with the StrainID Amplify kit according to manufacturer protocol and as previously described (Graf et al., 2021). Briefly, 10 µL of DNA and 10 µL of PCR mix (Shoreline Biome, United States) were added to each well with barcoded primers. The PCR reaction was carried out on a thermocycler (BioRad, Hercules, CA, United States) according to manufacturer protocol. Amplicons were screened on a QIAxcel (Qiagen, Germantown, MD, United States) Advanced system using the Fast Analysis protocol. Samples were then pooled together based on band intensity. Samples were cleaned using the GeneRead Size Selection Kit (Qiagen, United States) according to manufacturer protocol and resuspended in 50 µL of elution buffer. After verifying that both pools were pure, amplicons were pooled and sequenced on a PacBio Sequel IIe. Sequences were demultiplexed with SBAnalyzer, then split into groups of approximately 35 samples. DADA2 software (https://benjjneb.github.io/dada2/) was used to call ASVs, and taxonomy was assigned using the Athena database (Graf et al., 2021; Callahan et al., 2016). Data analysis was performed in QIIME2 and R (McMurdie and Holmes, 2013; Bolyen et al., 2019). Visualizations were made with GraphPad Prism and microViz (Barnett et al., 2021). The code used is available at https://github.com/brandon-osullivan/Code-for-Maltz-Matyschsyk-et-al-2022.

### Microsphere immunoassay for cytokines identification

Cytokines/Chemokine levels in the serum samples were measured in duplicate using the Luminex^®^ 200^™^ instrument and Milliplex^®^MAP kits from EMD Millipore (Cat #HSTCMAG-28SK, HCYTA-60K, HCYP2MAG-62K and HCYP4MAG-64K) according to manufacturer’s protocol. A 96 well plate provided with the kit was first washed with 200 µl per well of wash buffer. The wash buffer was then discarded, and 25 µl of serum sample was added to the plate in duplicate along with 50 µl of standards and controls as provided with each kit. Assay buffer (25 µl) was then added to the sample wells followed by 25 µl of premixed magnetic beads (provided with the kit) to each well. The plate was covered with a dark lid and placed on a plate shaker (200 rpm on a Barnstead 4625 Titer plate shaker) overnight at 4°C in a dark room. On the following day, the plate was washed 3X with 200 µl of wash buffer using BioTek ELx405^™^ microplate washer with magnetic capture: after washing, detection antibodies (50 µl) were added to each well and the plate was incubated for 1 h on a plate shaker covered with a foil. Streptavidin-Phycoerythrin (50 µl) was added to each well and the plate was incubated for 30 min on a plate shaker covered with foil. Finally, the plate was washed 3 × with 200 µl of wash buffer using BioTek ELx405^™^ microplate washer and analyzed using the Luminex^®^ 200^™^ instrument (Calibrated each week with Luminex 200 Calibration and Performance Verification kits: Cat # LX2R-CAL-K25, LX2R-PVER-K25) with 150 µl of Sheath Fluid present in each well. Standard curves were generated using the Luminex xPONENT^®^ software and the concentrations of cytokines/chemokines in the serum samples were calculated using these standard curves in pg/mL.

### Microsphere immunoassay for Ig response to Sars-CoV-2 epitopes

Specimens were assessed for the presence of antibodies reactive with SARS-CoV-2 using an MIA as previously described (Yates et al., 2021). Briefly, recombinant SARS-CoV-2 N protein antigen (Native Antigen Company, United Kingdom) and the receptor binding domain (RBD) of the SAR-CoV-2 spike protein (MassBiologics, MA, USA) were covalently linked to the surface of fluorescent microspheres (Luminex Corporation, TX, USA). Additional target antigens (S1 or S2 domain (Native Antigen, United Kingdom) of SARS-CoV-2 were included in the multiplexed microsphere assay. Serum samples (25 μl) and antigen-conjugated microspheres (25 μl) were mixed and incubated before washing and further incubation with phycoerythrin-conjugated antisera. The antisera used were chosen to specifically recognize, as indicated, total antibodies (pan-Ig), or, individually IgM, IgA, IgG, IgG1, IgG2, IgG3, IgG4. After washing, the microsphere fluorescence intensity (MFI) was quantified with a FlexMap 3D Luminex analyzer (Luminex Corporation, TX, United States). Results were either direct MFI values with reactivity based upon a defined cutoff MFI, or, were normalized by comparison to the MFI of the negative controls and expressed as the ratio between the two (P/N). Positive reactivity is determined by a result that is ≥6 SD above the cutoff; results that fall between 3 SD and 6 SD are considered “Indeterminate”.

### Optimization of biosensor chip

We set out to optimize the initial biosensor chip configuration by comparison to an established Luminex assay. First, we performed a dose response curve examining the limit of detection for the 11 analytes that were evaluated in both the GCFP assay and the Luminex data using parameters that had been employed with the first-generation GCFP chip. Chips were spotted as described above with the capture antibodies for the following analytes: TNF-α, IL-7, IL-6, IL-4, IL-21, IL-2, IL-1β, IL-17A, IFN-δ, CCL-3, CCL20. Recombinant proteins (R&D Systems, MN, USA; Shenandoah Biotechnology, Inc, PA, United States) were then diluted to 5 ng/mL, 1 ng/mL, 200 pg/mL, and 1 pg/mL and recirculated over the chip as described above. Detection ratios were calculated, and the limit of detection was determined for each matched pair set by GCFP. We then optimized the capture and secondary antibodies to reach values detectable with the Luminex MIA assay.

## Results

### Identification and validation of regions of interest

Since MIS-C and COVID-19 are relativity new disease states, we set out to develop our first generation GCFP biosensor chip based on literature available early in the pandemic (Cabrero-Hernandez, 2020; Bar-Meir et al., 2021; Consiglio et al., 2020; Ramaswamy et al., 2020; Whittaker et al., 2020; Buszko et al., 2021; Filbin et al., 2021). These studies showed an array of cytokines, chemokines, tissue damage markers, and inflammatory indicators that differed significantly in children with MIS-C, COVID-19, and Kawasaki (Table 1). Our goals with the first-generation chip were to: 1) identify commercially available matched pair antibodies for our ROIs to differentiate MIS-C from COVID-19, Kawasaki, and healthy controls and 2) develop a GCFP assay to simultaneously evaluate if these markers could be used as a disease-specific biosignature. We validated 42 commercially available matched pair antibodies by ELISA using recombinant proteins (Supplementary Figure S1). Nine commercially available kits that did not meet our ELISA standards when performed following manufacture protocols, and were omitted from further analysis (Supplementary Figure S1).

### Development of the grating-coupled fluorescence plasmonic biosensor chip assay

**FIGURE 2:**
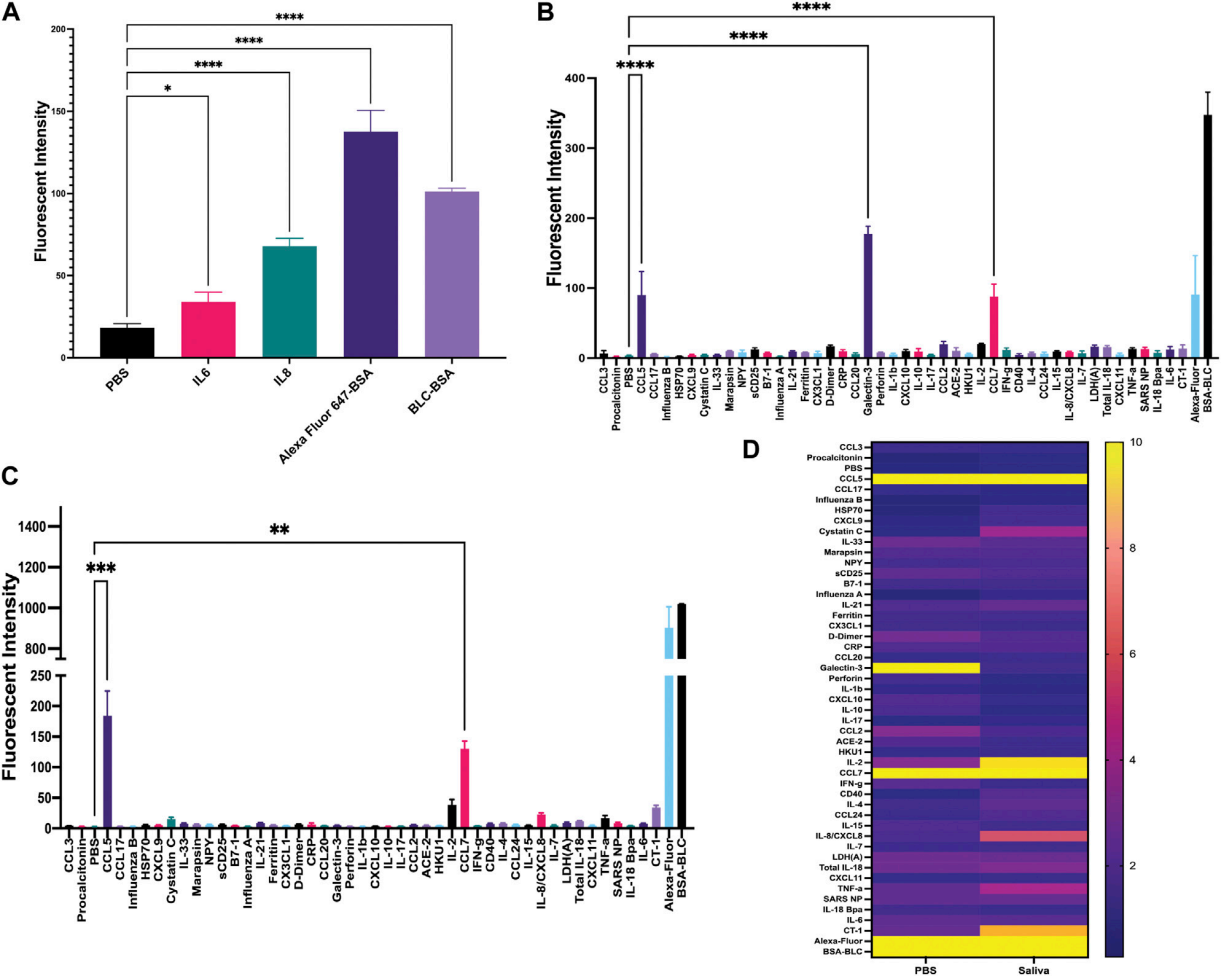
Development of sandwich-based ELISA assay on a gold-coated nanoscale grating surface chip. **(A)** Capture antibodies for IL-6 and IL-8 were immobilized onto the chip, along with the negative control (PBS) and two positive controls; biotin-BSA (BLC-BSA, to show streptavidin binding) and Alexafluor 647-BSA (to show stabilization during washing), 20 ng/mL of recombinant IL-6 and IL-8 were flowed over the chip. The fluorescence intensity was collected as previously described. Fluorescent intensity within the ROIs for IL-6, IL-8 and positive controls was significantly higher than the negative control. **(B)** Forty-two capture antibodies were immobilized onto the chip, 20 ng/mL each of recombinant CCL5, galactin-3 and CCL7 diluted in PBS was run over the chip and the fluorescence intensity was collecting for each ROI as previously described. The fluorescent intensity within the ROIs for CCL5, galactin-3 and CCL7 were significantly higher than the negative control. **(C)** Forty-two capture antibodies were immobilized onto the chip, 20 ng/mL of recombinant protein CCL5 and CCL7 diluted in human saliva was run over the chip and the fluorescence intensity was collected for each ROI as previously described. The fluorescent intensity within the ROIs for CCL5 and CCL7 were significantly higher than the negative control. **(D)** A heat map was generated using the detection ratio, which was normalized to the negative control to compare data in B and **(C)**. One-way ANOVA analysis was performed (* = *p*< 0.05, ** = *p*< 0.01, *** = *p*< 0.001, **** = *p*< 0.0001. The data are presented as the average of five ROIs for each analyte ( ± standard error of the mean).

Once matched-pair kits were validated using ELISA, we then tested their ability to perform a sandwich-based immunoassay on the gold-coated nanoscale grating surface chip using IL-6, IL-8 and both positive (Alexafluor-647 and biotin-BSA) and negative (PBS) controls (Figures 1, 2A). When compared to PBS (negative control), the fluorescent intensity within the ROIs for IL-6, IL-8 and positive controls was significantly higher, indicating 20 ng/mL of IL-6 and IL-8 recombinant proteins could be detected with GCFP technology. Next, we examined the specificity of the ROIs by spotting all the capture antibodies in Table 1 onto the chip and recirculating 20 ng/mL of recombinant CCL5, galactin-3 and CCL7 diluted in PBS over the biosensor chip (Figure 2B). When compared to PBS (negative control ROIs), the fluorescent intensity within the ROIs for CCL5, galactin-3 and CCL7 were significantly higher, indicating that the GCFP assay was able to capture and detect these recombinant proteins at cognate ROIs and that there was no significant cross-reactivity between these recombinant proteins and unrelated ROIs. To evaluate the use of human salvia as the sample matrix, CCL5 and CLL7 (20 ng/mL) were spiked into commercially available pooled human saliva (Innovative Research, MI, United States) (Figure 2C). Spiked samples in pooled saliva matrix produced higher values than when the same analytes were diluted in PBS owing to the presence of endogenous CCL5 and CCL7 in the saliva pool. A heat map was created using the detection ratio for each set of 5 ROIs (Figure 2D). The heat map shows the detection ratio and indicates positive detection of all recombinant proteins when normalizing to the negative control ROIs on the same chip.

### Analysis of patient samples by grating-coupled fluorescence plasmonic imaging

**FIGURE 3:**
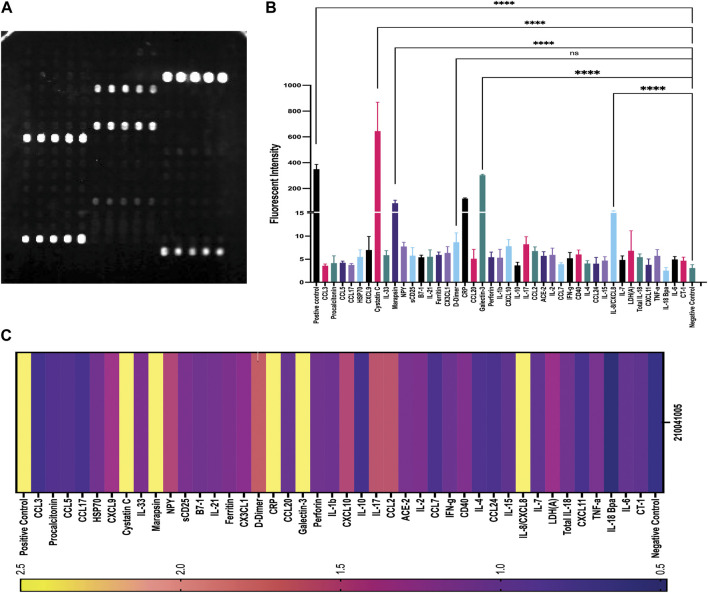
Example of patient (210041005) data generated from GCFP assay. **(A)** Forty-two unique capture antibodies were immobilized on regions of interest in sets of 5 ROIs on the chip and patient sample number 210041005 was run over the chip and an image of the GCFP sensor chip output was captured. **(B)** Fluorescent intensity within the ROIs were collected as previously described, detecting cystatin C, Marapsin, D-dimer, galaectin-3, and IL-8. **(C)** A heat map of the detection ratio was generated and shows detection of the same analytes as in **(B)**. One-way ANOVA analysis was performed (* = *p*< 0.05, ** = *p*< 0.01, *** = *p*< 0.001, **** = *p*< 0.0001. The data are presented as the average of five ROIs for each analyte ( ± standard error of the mean).

**FIGURE 4:**
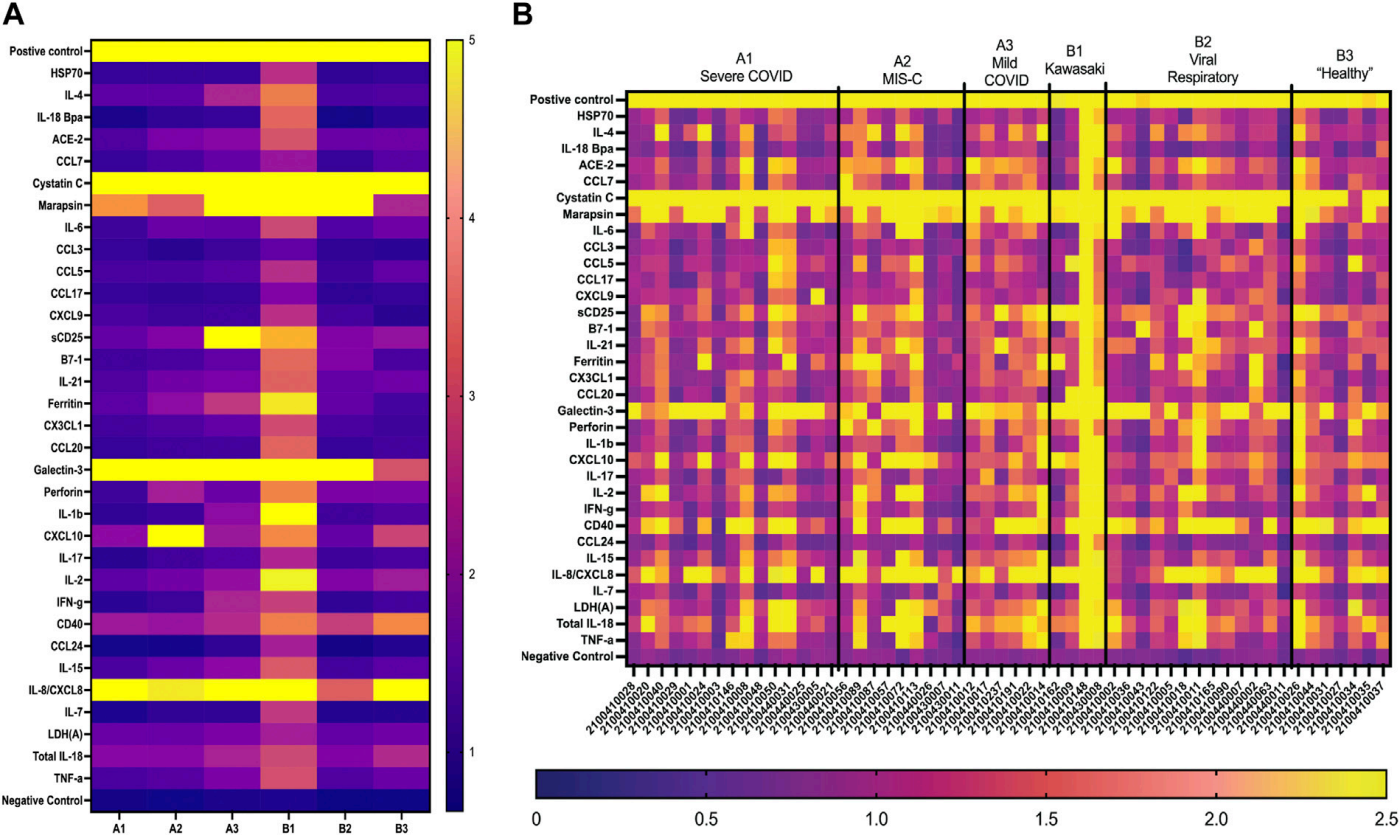
GCFP detection ratio for patient saliva samples. Saliva samples obtained from each of the patient cohorts; 54 samples (A1 = 15, A2 = 9, A3 = 6, B1 = 4, B2 = 13, B3 = 7) were run over the first-generation biosensor chip. **(A)** Heat maps generated for each cohort using the mean detection ratio for each analyte, showing candidate biomarkers for the A2 (CXCL10), A3 (sDC25) and B1(IL-1β and IL-2) cohorts. **(B)** Heat maps generated for each individual patient demonstrate individual variation in disease state.

**FIGURE 5:**
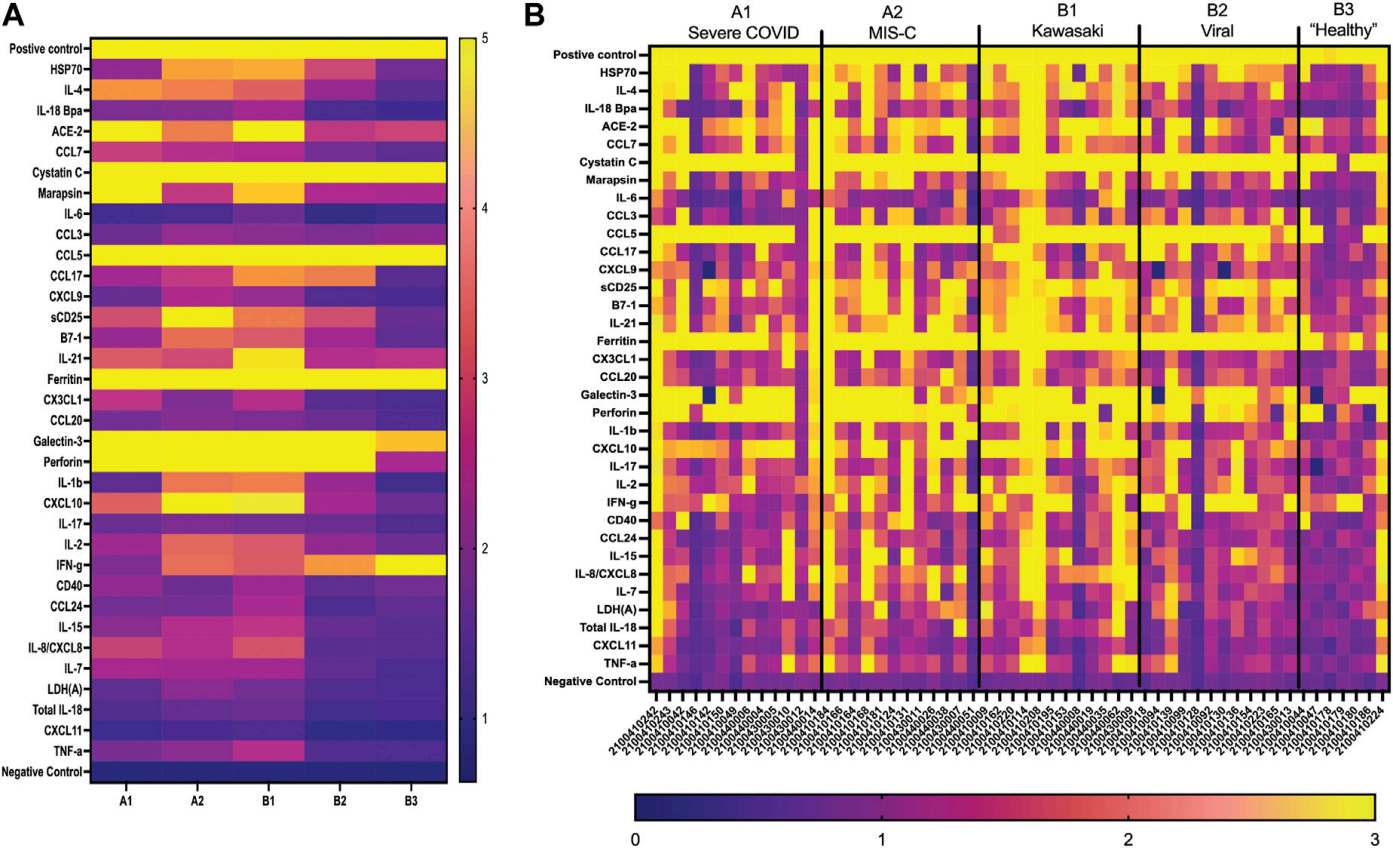
GCFP detection ratio for serum samples from patient cohorts. Serum samples from each of the cohorts were tested on the first generation GCFP biosensor chip. 56 samples (A1 = 13, A2 = 12, B1 = 12, B2 = 12, B3 = 7) were tested. **(A)** Heat maps generated for each cohort using the mean detection ratio for each analyte, showing a more robust signature per cohort then within saliva. **(B)** Heat maps generated for each patient, showing individual variation in disease state.

The first-generation GCFP chip was then evaluated to identify a candidate biomarker signature of disease using data from each of our 6 cohorts. Patient saliva and serum were analyzed with the biosensor chips and the fluorescent intensities were captured using GCFP reader (Ciencia, Inc., CT, United States) (Figure 1). For each patient sample, an image was produced from the reader; Ciencia software was used to identify ROIs, and then the fluorescent intensities for each ROI were measured and the corresponding detection ratios calculated. Figure 3 shows representative saliva data generated from a randomly selected patient from cohort B2. The image generated from patient ID 210041005 shows that five capture antibodies and the two positive controls (Biotin-BSA and alexafluor-647 labeled BSA) have a positive signal (Figure 3A). Cystatin C, Marapsin, CRP, Galectin-3, and IL-8 ROIs were each significantly different from the negative control, indicating that these analytes are present at high levels in this patient’s saliva (Figure 3B). The fluorescent intensities were then normalized to the negative control and a heat map was generated to visualize the biomarker signature (Figure 3C). Cystatin C, Marapsin, CRP, Galectin-3, and IL-8 all had high detection ratios. Saliva from the six cohorts (A1 = 15, A2 = 9, A3 = 6, B1 = 4, B2 = 13, B3 = 7) were similarly analyzed, and results are displayed as heatmaps (Figures 4A,B). Figure 4A shows the mean for each analyte from all the patient samples in each of the 6 cohorts. From this initial small data set we can begin to identify potential biomarkers for A2 (CXCL10), A3 (sDC25) and B1(IL-1 β and IL-2). Interestingly, when comparing individual patients from each cohort we can see variation in these biomarker signatures, suggesting variation in disease state presentation (Figure 4B). From the serum cohorts, 56 samples (A1 = 13, A2 = 12, B1 = 12, B2 = 12, B3 = 7) were analyzed with the first-generation biosensor chip configuration and heat maps were generated for each group and for each individual patient (Figure 5A) shows the mean for each analyte from all the patient samples run in each of the 5 cohorts and indicates potential biomarker signatures for each cohort. In A2 (MIS-C group) biomarker sCD25 was found to be a potential unique biomarker for this group, while IL-21 was a unique biomarker for B1 (Kawasaki). Again, variation between individuals’ biomarker signatures within the different cohorts indicates heterogeneous disease presentation (Figure 5B).

### Interrogation of grating-coupled fluorescence plasmonic saliva outliers using 16S-23S rRNA gene amplicon sequence analysis

**FIGURE 6:**
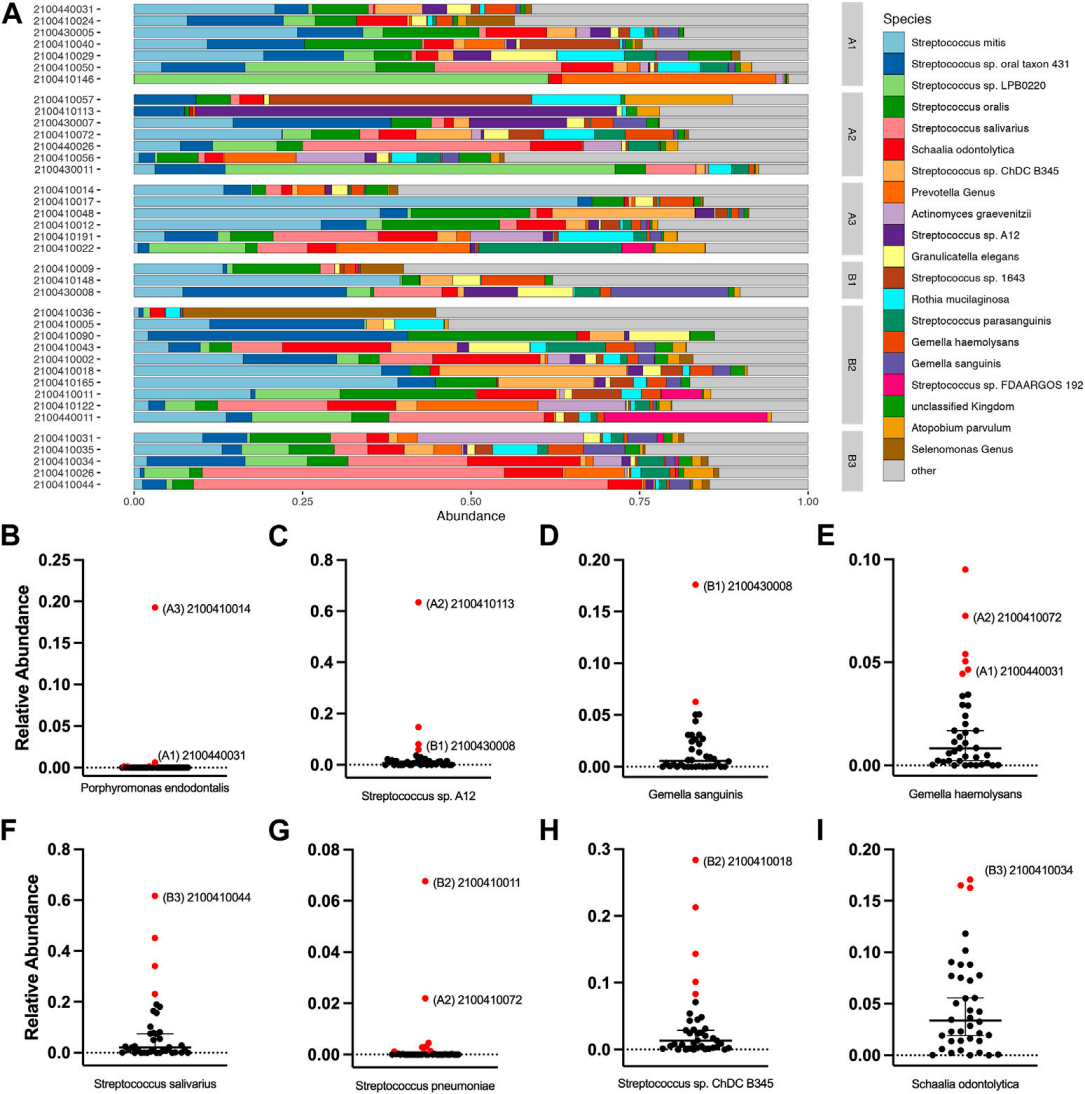
Salivary microbiome of samples analyzed using GCFP. From saliva samples analyzed by GCFP using the first generation GCFP biosensor chip, 34 samples were also analyzed by 16S RNA high throughput sequencing. Relative abundance at species level was calculated. **(A)** Relative abundance of the top 20 most abundant taxa across all samples. **(B–I)** Plots of the relative abundance for 8 bacterial species that were elevated in one or more outlier samples from analyte data. Error bars represent a 95% confidence interval of the median. Points in red were identified as outliers by the ROUT method. Samples that were analyte outliers and had an increase in that species are labeled with their cohort.

We noted some analyte outliers within individual saliva samples (Figure 4B). To further analyze the saliva from these samples, we performed 16S-23S’ rRNA PacBio sequencing. For this study, we singled out sequencing data for the samples that were run over the first-generation biosensor chip and compared the relative abundances of different bacterial species. To identify bacterial species that were more abundant in the samples that were analyte outliers, we applied a 99% confidence interval, and confirmed outliers with the ROUT method. These data revealed a difference in relative abundance of several bacterial species, some of which could be linked to immunocompromised status, oral hygiene or other diseases (Figure 6). For example, sample 2100410014 from cohort A3 had an inflammatory profile that more closely resembled a sample from the MIS-C or Kawasaki cohorts. This sample also had highly elevated levels of *P. endodontalis*, a known oral pathogen, with a relative abundance of 19.26% of the overall community (median = 0.0%) (Figure 6B). In sample 2100410113, another saliva sample that was an outlier in the biomarker analysis, we found that the community was composed primarily of *Streptococcus* sp. A12 (63.45%, median = 0.74%) (Figure 6C). Sample 2100430008 had high levels of *Gemella sanguinis* (17.61%, median = 0.59%) and *Streptococcus* sp. A12 (8.00%, median = 0.74%) (Figures 6C,D). Sample 2100440031 had raised levels of *Porphorymonas endodontalis* (0.64%, median = 0.0%) and *G. haemolysans* (4.65%, median = 0.84%) (Figures 6B,E). In sample 2100410044, the majority of the community was *Streptococcus salivarius* (61.67%, median = 2.05%) (Figure 6F). Sample 2100410011 had elevated levels of *Streptococcus pneumoniae* (6.77%, median = 0.0%) (Figure 6G). 2100410072 also had elevated levels of *S. pneumoniae* (2.20%, median 0.0%), as well as above average levels of *Gemella haemolysans* (7.26%, median = 0.84%) (Figures 6E,G). In sample 2100410018, we observed elevated levels of *Streptococcus* sp. ChDC B345 (28.39%, median = 1.34%) (Figure 6H). Our last GCFP analyte outlier, sample 2100410034, had elevated levels of *S. salivarius* (18.09%, median = 2.05%) and *Schaalia odontolytica* (17.07%, median = 3.36%) (Figures 6F,I).

### Refining the composition of a second-generation chip using Microsphere Immunoassay

**FIGURE 7:**
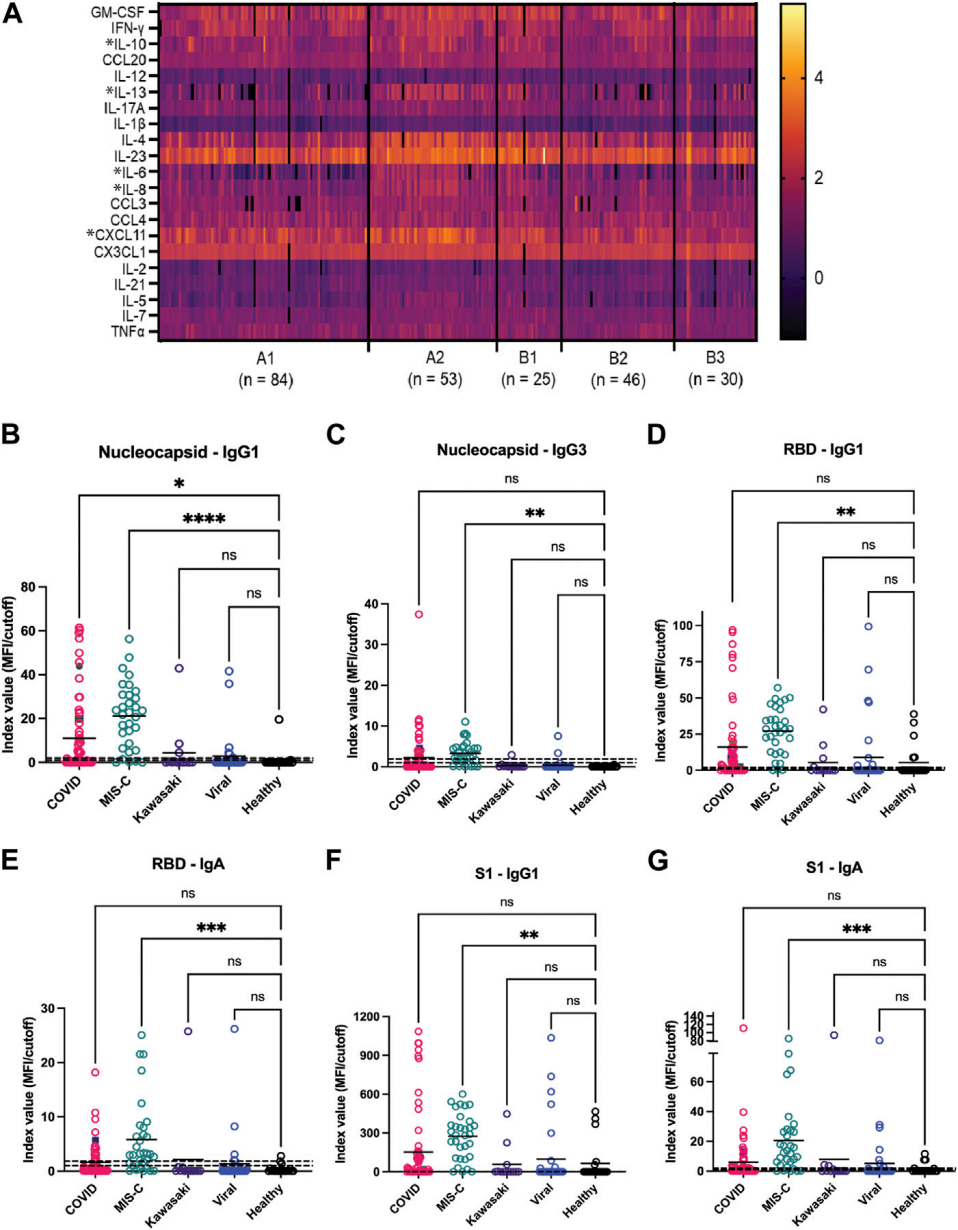
Potential analytes found by MIA can inform the evolving composition of a second generation GCFP biosensor chip. **(A)** A heat map was generated from microsphere immunoassay from serum samples for each cohort. * Indicates a significant difference in that cytokine for the A2 cohort for CXCL11, IL-8, IL-6, IL-4, IL-13, & IL-10 analytes. **(B–G)** The antibody response to the SARS-CoV-2 nucleocapsid and spike components (full spike, RBD, S1, S2), the MIS-C cohort had a significantly higher IgA response to SARS-COV-2 spike domains as compared to the COVID-19 cohort. One-way ANOVA analysis was performed (* = *p*< 0.05, ** = *p*< 0.01, *** = *p*< 0.001, **** = *p*< 0.0001.

Concurrent with these microarray studies, candidates for inclusion on a second generation GCFP sensor chip were evaluated. We examined 249 serum samples (180 from the United States and 69 from Colombia) from five different cohorts (A1, A2, B1, B2, and B3). Patient samples were analyzed for levels of 26 cytokines/chemokines using the Luminex xMAP^®^ multiplex assay. IL-4, IL-6, IL-10, and IL-13, known to be involved in Th_2_ type immune response and promoting B cell differentiation to plasma cells, were significantly elevated in the A2 (MIS-C cohort) when compared to most other cohorts (Figure 7A). Furthermore, chemokines IL-8 and CXCL11 were also found to be expressed at higher levels in the A2 cohort. By comparison, the first-generation GCFP chip detected higher IL-8 for cohorts A1, A2, B1 and IL-4 in cohorts A1 and A2 (Figure 5A), while it did not detect IL-6 for any of the cohorts. It is important to note that MIA may use different proprietary antibodies or conditions or recognize different epitopes on these targets. The results from the Luminex studies were representative of a higher sample size than the GCFP analysis, which may also contribute to differences in the results. MIA data did confirm IL-10, IL-13 and CXCL11 as possible new targets for the second-generation chip.

The antibody response to the SARS-CoV-2 nucleocapsid and spike components (full spike, RBD, S1, S2) were measured using a MIA to separately detect anti-viral IgM, IgA, and all four IgG subclasses. Figures 7B–G shows that responses in the MIS-C cohort were substantially different from standard COVID-19 infections and from healthy controls. Note that some sera in the control cohort had antibodies to either the spike proteins or nucleocapsid, indicating previously undetected SARS-CoV-2 infection or an unreported vaccination. The predominant IgG subclasses produced in response to COVID-19 infection are IgG_1_ (Yates et al., 2021; 2021); both the COVID-19 and MIS-C cohorts had prominent IgG_1_ response centered around the RBD/S1 components of SARS-CoV-2 spike protein. The MIS-C cohort generally made a stronger IgG_1_ response as compared to the COVID-19 cohort. Notably, the MIS-C cohort made a significantly higher IgA response to SARS-COV-2 spike domains as compared to the COVID-19 cohort (Figure 7G), indicating SARS-COV-2 spike antigen as a possible target for the second generation GCFP chip.

### Comparison of grating-coupled fluorescence plasmonic biosensor chip biomarker signature to Cytokine Microsphere Immunoassay results

**FIGURE 8:**
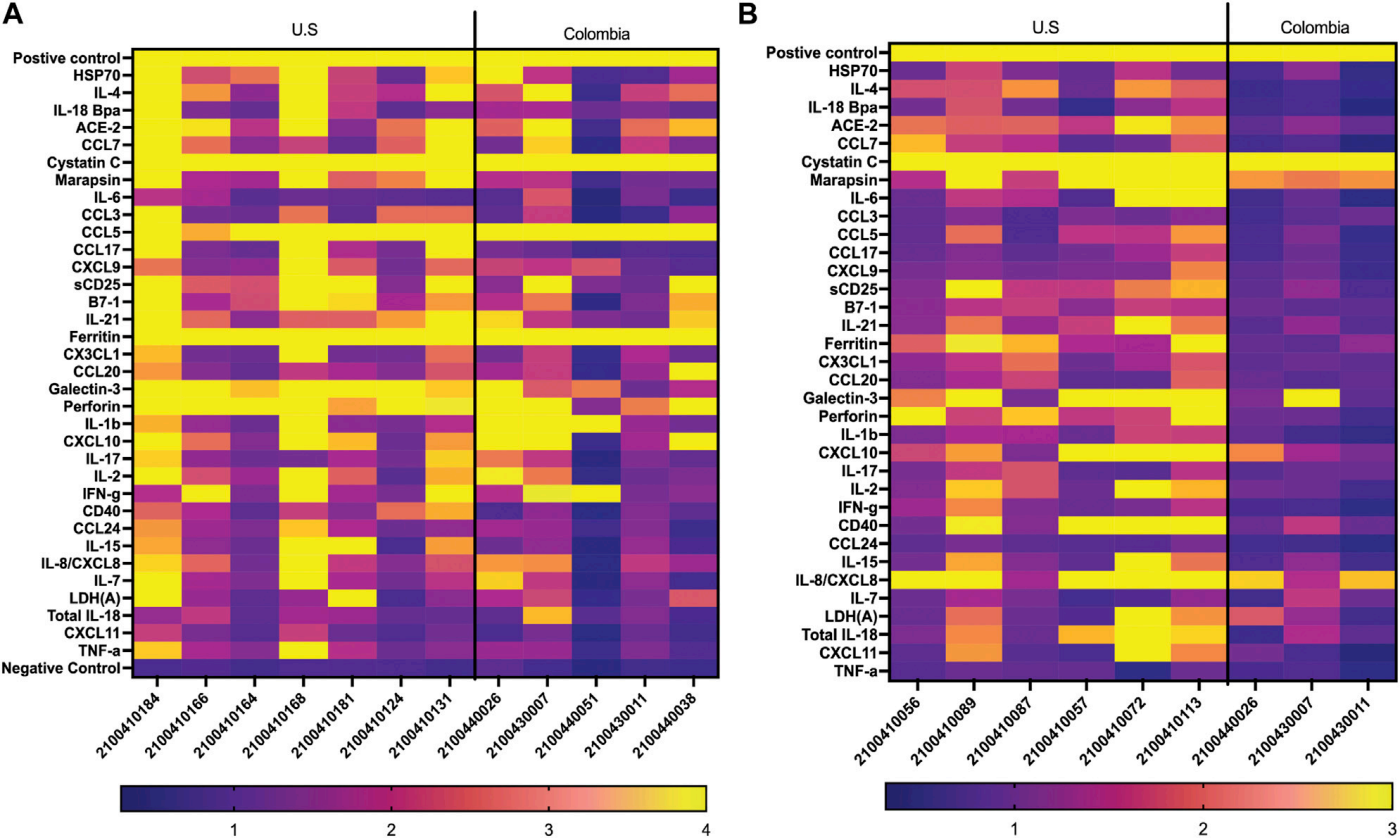
Comparison of USA vs. Colombia MIS-C patient samples by GCFP microarray. **(A)** Heat map of A2 serum samples comparing USA to Colombia samples. The USA patient population has a more robust signature. **(B)** Heat of A2 saliva samples comparing USA to Colombia samples, USA has a more robust signature.

We compared US serum samples with Colombia serum samples using both GCFP biomarker signature data and cytokine microbead assay (Figure 8A; Table 2). In cohort A2 (MIS-C), the biomarkers detected by the GCFP chip in US serum samples produced a more complex and robust signature when compared to the Colombia serum samples (Figure 8A). We also compared US patient saliva samples with Colombia saliva samples: biomarker signatures produced by GCFP microarray had a trend similar to that observed with serum samples (Figure 8B). Correspondingly, MIA performed on serum samples showed lower levels of cytokine in Cohort A2 Colombia samples compared to US samples (Table 2). Taken together these data indicate a difference in immune response between these two populations.

**TABLE 2:** MIA Cytokine levels US vs. Colombia.

	A1		A2		B1		B2	
**Cytokine (pg/mL)**	US	Colombia	US	Colombia	US	Colombia	US	Colombia
**CXCL11**	262.8	359.2	1123	520.2	379.3	466.5	181	195.6
**GM-CSF**	120.6	239.4	203.1	163.2	250.3	291.4	111.6	228.7
**CX3CL1**	236.1	246.3	272	254.2	246.9	268.3	213.6	244.9
**IFN-γ**	105	113.1	137.3	150.4	92.00	87.81	56.72	106.5
**IL-10**	46.3	174.6	187.4	157	71.07	79.41	26.82	75.5
**CCL20**	33.2	49.1	59.7	151.4	43.72	64.38	27.83	42.6
**IL-12(p70)**	5.3	7.6	5.2	5.9	6.6	5.2	6.052	6.3
**IL-13**	34.1	14.03	119.2	24.53	114.4	20.26	19.98	27.4
**IL-17A**	21.9	24.18	26.2	18.50	27.45	21.86	15.84	25.8
**IL-1β**	3.7	4.1	5	4.3	5.4	4.3	3.779	4.3
**IL-2**	6	7.4	6.8	6.4	6.9	7.1	4.4	7.3
**IL-21**	7.1	9.2	10.1	7.1	10.17	11.80	7.2	11
**IL-4**	250	71.05	689.4	125.6	532.0	106.6	146.0	129
**IL-23**	1103	792.4	1302	768.8	1530	43,016	532.5	1031
**IL-5**	8.4	6.068	19.4	7.8	20.49	6.8	24.93	6.6
**IL-6**	27.3	8.9	77.0	150.4	61.24	12.35	23.08	11.28
**IL-7**	15	14.14	18.5	15.5	18.59	30.50	13.68	14.66
**IL-8**	39.6	18.3	104	35.9	76.84	29.86	24.11	29.98
**CCL3**	32.7	43.86	48.5	26.4	37.11	28.67	73.93	32.95
**CCL4**	45.7	54.39	64.8	47.61	58.97	61.19	44.98	69.87
**TNF-α**	16.5	30.7	33.4	35.2	27.31	33.32	19.92	33.33
**IFN-α2**	124.7	227	214	153.6	267.7	183.5	465.2	234.5
**IL-15**	27.8	17.3	26.6	11.94	16.58	6.47	22.66	9.795
**IL-18**	270.7	362.7	835	1846	599.4	507.3	574.5	557.6
**IL-33**	422.8	85.4	831.4	163.2	930.3	712	355.5	250.4
**IFN-β**	1421	527	3446	468	3158	156.8	2750	647.4

**FIGURE 9:**
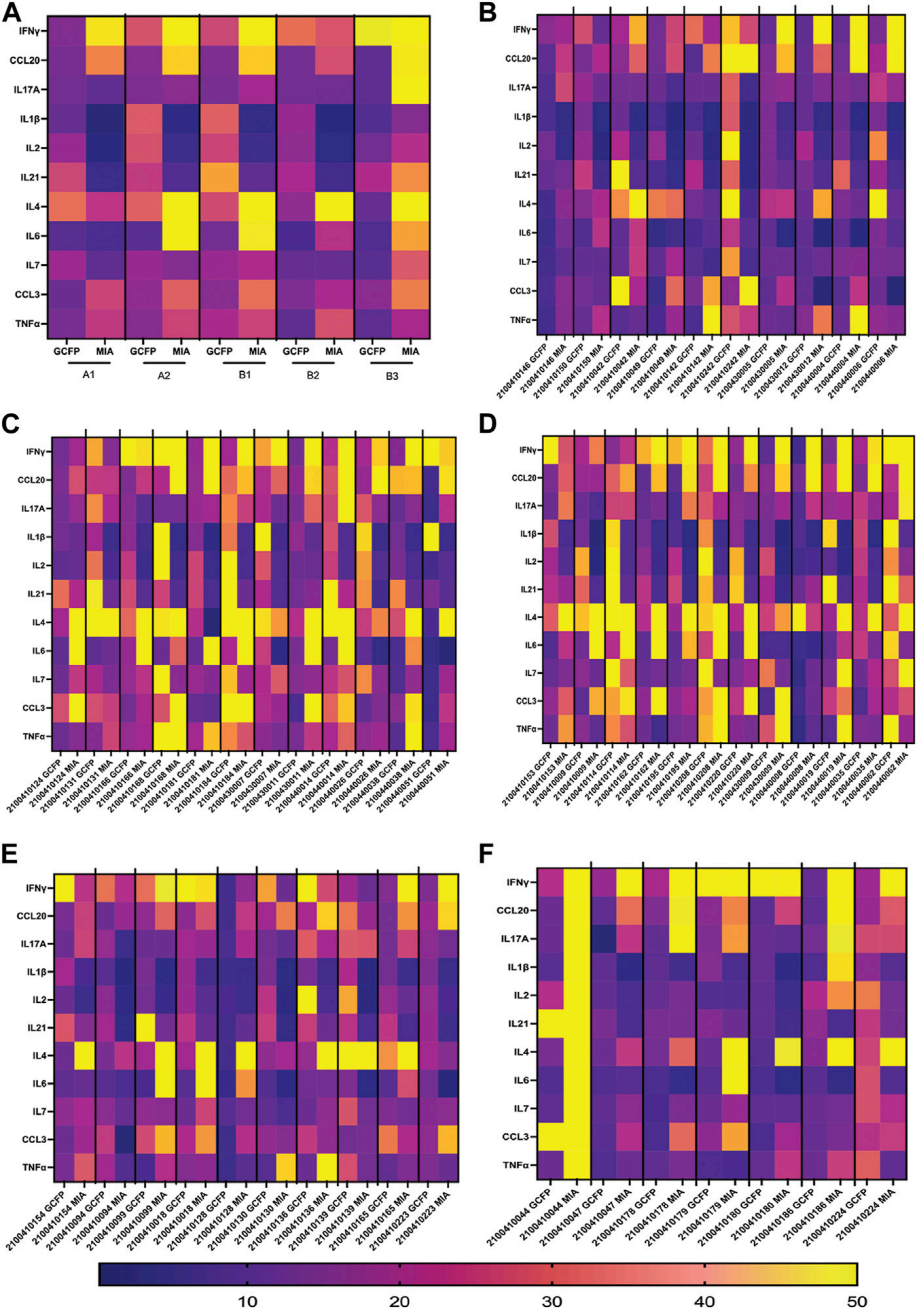
Serum from Cohorts and individual patient comparison of GCFP microarray assay to MIA. To compare a traditional MIA with the GCFP microarray, 11 analytes were included in both assays. **(A)** A heat map was generated by log transforming GCFP detection ratios and using the mean of each analyte detection ratio for each cohort. Of the 11 analytes, 5 were below the detection limit of the GCFP first generation biosensor chip. **(B)** A heat map generated by log transforming GCFP detection ratios from individual serum samples from cohort A1. **(C)** A heat map generated by log transforming GCFP detection ratios from individual serum samples from cohort A2. **(D)** A heat map generated by log transforming GCFP detection ratios from individual serum samples from cohort B1. **(E)** A heat map generated by log transforming GCFP detection ratios from individual serum samples from cohort B2. **(F)** A heat map generated by log transforming GCFP detection ratios from individual serum samples from cohort B3. 16 of the 44 shared serum samples had similar biomarker signatures, while IL-4, IL-6 and CCL20 were not consistently detected on the GCFP chip.

**FIGURE 10:**
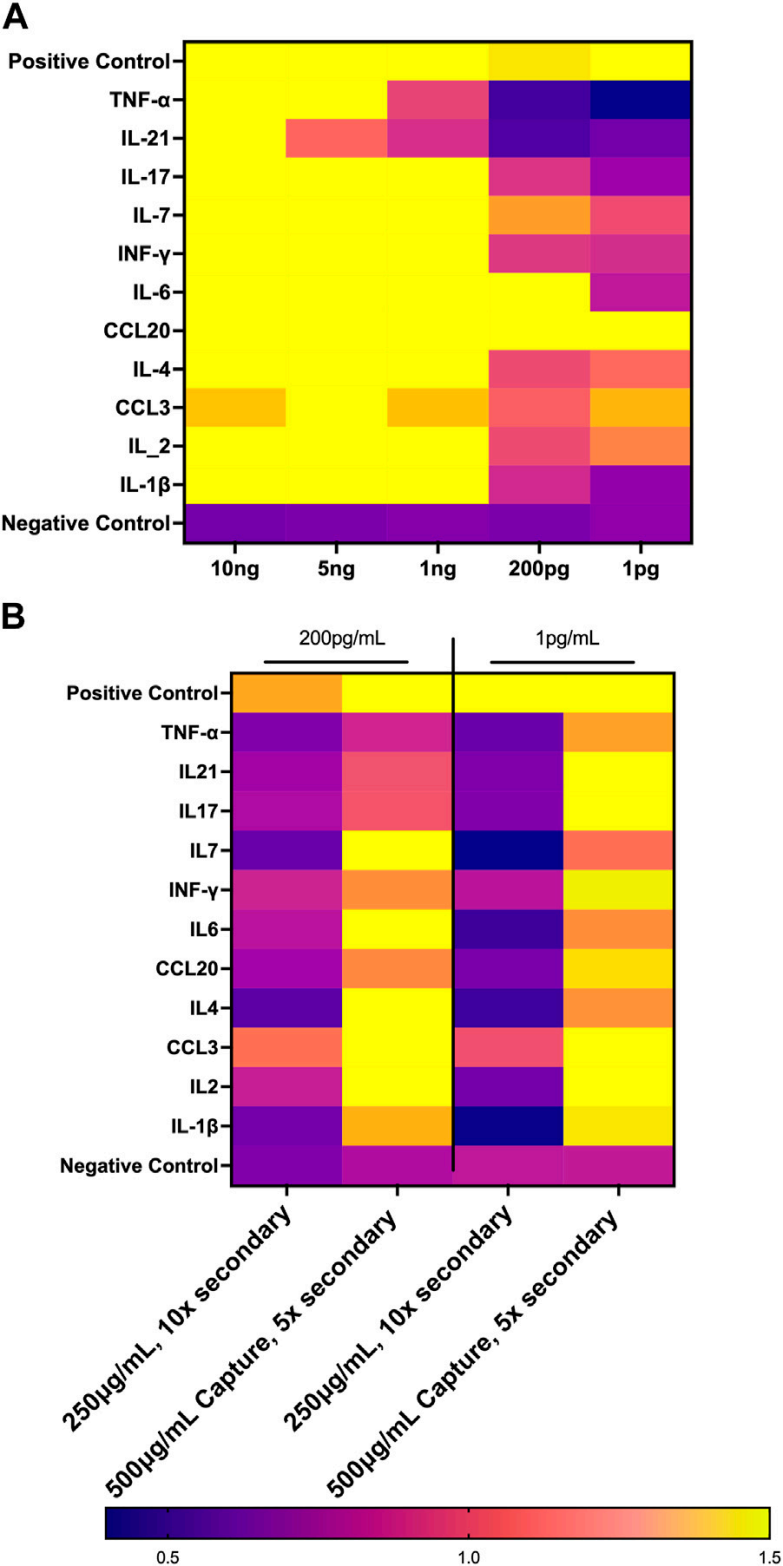
Optimization of First Generation GCFP Chip. **(A)** Measurements of the limit of detection for 11 analytes from the first-generation biosensor chip. 1 ng/mL is the limit of detection for most analytes in this assay. **(B)** Optimizing reagents can increase the limit of detection.

To compare MIA data directly with the biomarker signature generated on the first-generation chip, 11 analytes that had been included in both assays and detection ratios were log transformed to visualize the data by cohort and as individual patients (Figure 9). The heat map reveals that most of the analytes were detected within both biomarker signatures generated from MIA and GCFP for the cohorts with the exception of IL-4, IL-6, CCL20 and IL-21. When we compare individual samples, similar signature patterns were found in the following samples for both MIA and GCFP; 2100410146, 2100410150, 2100410042, 2100410049, 2100410131, 2100410168, 2100410184, 2100440014, 2100410153, 2100410114, 2100410208, 2100440008, 21004400062, 2100410094, 2100410139, and 2100410165. When noting the quantified analyte levels (pg/mL) in the microbead assay dataset (Supplementary Table S1) it also becomes clear that some of the analytes (IL-4, IL-6 and CCL20) were not detected on the GCFP chip while others, like IL-1 β, were detected on the chip but not within the microbead assay. To address this, we performed a dose response curve on the first-generation chip for analytes that were measured both by MIA and GCFP using PBS that was doped with different amounts of recombinant proteins (10ng, 5ng, 1ng, 200pg, 1 pg/mL) (Figure 10A). These data indicate that for many of the analytes analyzed by both MIA and GCFP, the first-generation chip has a detection limit of 1 ng/mL while MIA could detect levels as low as 1 pg/mL, which could explain the analytes that were undetected by GCFP. We further optimized the GCFP chip to improve the assay’s limit of detection (Figure 10B). We first increased the amount of secondary antibody to 10-fold the amount recommended by the manufacturers for ELISA. We did not see an improvement with this change (Figure 10B). We then increased the amount of capture antibody immobilized on the GCFP chip by twofold (500 μg/mL) and we were able to see an improvement in limit of detection for most analytes, indicating that manipulating capture reagents can further improve detection limits. Future studies for the next-generation chip will not only be focused on adding other reported analytes now known to be important for MIS-C, but also optimizing each analyte reagent set to allow for detection in the pg/mL range.

## Discussion

Clinicians need a rapid, accurate test for MIS-C to guide treatment decisions. Here we show that using GCFP technology we can characterize two biomarker signatures within two different biofluids (serum and saliva), one for a group/cohort and one for an individual patient. In saliva, the GCFP biomarker analytes found in all disease state cohorts were Cystatin C, Marapsin, Galactin-3, and IL-8. When designing the chip we included Cystatin C for its potential as being predicative of renal injury and due to its observed elevated levels in COVID-19 patients (Li et al., 2020). We were able to detect Cystatin C in all our cohorts; only one patient did not produce detectable Cystatin C. The normal range of Cystatin C is 10–12 mg/mL in saliva and, 0.63–8.0 mg/mL in serum; and has been shown to be upregulated in people with poor oral hygiene (Lie et al., 2001; Dickinson, 2002; Ziegelasch et al., 2019). This suggested that Cystatin C may present at levels exceeding the upper limit of GCFP detection under the conditions tested. Marapsin/pancreasin is a trypsin-like serine protease that has been described in pancreatic tissue; little is known about its role in other systems although it can be expressed in stratified squamous epithelium tissues (esophagus, cervix and larynx) (Bhagwandin et al., 2003; Li et al., 2009). We included this analyte to potentially predict pancreatic health since reduced pancreatic function increases risk of developing severe COVID-19 (Abramczyk et al., 2022). Our biosensor chip was able to detect marapsin in all of the infected cohorts (A1-3 & B1-2), but not in the healthy cohort (B3). Interestingly, marapsin has been found to be upregulated in the epidermis of patients with psoriasis and/or regenerating wound infections, suggesting a possible role in infection (Li et al., 2009). Galectin-3 is a ß-galactoside binding lectin that can drive neutrophil chemotaxis, bind TLR4, and increase production of pro-inflammatory cytokines during viral infections (Joo et al., 2001; Filer et al., 2009; Henderson and Sethi, 2009; Burguillos et al., 2015). Increased serum levels of galectin-3 (>30 ng/mL) have been correlated with severe COVID-19 outcomes (Eduardo et al., 2022). Our biomarker signature showed galectin-3 was present in all cohorts, while lower in healthy controls, indicating a role in infection. Galectin-3 may be upregulated in COVID-19 cohorts (A1,2,3) but the amount found in saliva were near saturation for the GCFP assay as configured. Finally, IL-8 cytokines are produced by a wide range of cells including oral keratinocytes and are a potential biomarker for predicting oral disease (Finoti et al., 2017; Lopez-Labady et al., 2021). IL-8 was also reported early in the pandemic to be a sensitive serum biomarker in both mild and severe COVID-19 patients (Li et al., 2020). Interestingly, GCFP detected salivary IL-8 in all cohorts, while in serum it was detected only in the A1, A2 and B1 cohorts.

There were some cohort-unique saliva biomarkers contributing to the biomarker signature from each of the different cohorts. GCFP detection of CXCL10 was only seen in Cohort A2 (MIS-C). CXCL10 is a chemokine that is produced by many different cell lines and tissues (Jong-Ho Lee et al., 2020). This chemokine has been identified in several studies as a contributing factor to the modulation and intensity of inflammation caused by SARS-CoV2 and recently was shown to be at higher levels in MIS-C patients (Callahan et al., 2021; Caldarale et al., 2021). sCD25 was solely detected on the GFCP chip in sera from cohort A3 (mild COVID). sCD25 is the soluble form of IL-2R alpha chain, has been linked to T-cell proliferation and inflammatory disease, and is a driver of disease pathogenesis (Russell et al., 2012). sCD25 was reported to be upregulated in the serum of pediatric patients with SARs-CoV2 infections and MIS-C (Mostafa et al., 2022). It is interesting to note that sCD25 is present in the saliva from the A3 cohort and present in the serum biomarker signature from A1, A2, and B1 cohorts. In Kawasaki patient serum, sCD25 has been found to be 3-100x higher than in healthy controls, which is consistent with our observations (Teraura et al., 2016). In cohort B1, we observe the most diverse saliva biomarker signature when compared to the other cohorts, with ferritin, IL-1β and IL-2 being uniquely found at elevated levels in this cohort. Studies have indicated that high serum levels of ferritin are present in Kawasaki patients and ferritin is a predictor of non-responsiveness to intravenous immunoglobulin (IVIG) therapy (Yamamoto et al., 2015; Kim et al., 2021; Qiu et al., 2022). IL-1β has previously been reported to play a key role in the inflammatory profile of Kawasaki disease, and IL-2 is significantly higher in children with Kawasaki than in healthy controls (Okada et al., 2003; Hoang et al., 2014; Alphonse et al., 2016). These findings indicate that data obtained by GCFP in saliva samples are consistent with recent scientific studies and show potential for this assay as a diagnostic tool. We showed that there was variation between individual saliva samples in patients within the same cohort, indicating differences in immune responsiveness.

The microbiome 16S RNA data helped to decipher potential reasons for some of the observed differences within cohorts by demonstrating imbalances in the microbial community or the presence of oral pathogens. In future studies, we plan on designing an approach to detect these imbalances and/or pathogens by using an oligonucleotide based GCFP biosensor chip to characterize the oral microbiome. Ultimately, it will be interesting to explore the possibility of doing both protein capture, antibody capture, and oligonucleotide analyte capture on the same sensor chip to further improve the efficiency and versatility of the assay.

Serum samples demonstrated a more complex biomarker signature than saliva samples, on both individual and inter-cohort levels (Figures 4, 5), which could be due to increased analyte presence in serum compared to saliva, better preservation of analyte in serum, or the choices of analytes included on the first-generation chip. The salivary proteome has 3,074 unique human proteins and only shares 1,234 proteins with blood plasma (https://www.salivaryproteome.org). GCFP biomarker analytes found in all disease state cohorts were cystatin C, ferritin, galectin 3, and perforin. As noted above, cystatin C, galectin-3 and ferritin are all found in serum at levels >250 ng/mL which could be at the limit of saturation for these assays. As expected, perforin, a glycoprotein responsible for pore formation in cell membranes of target cells, which plays an important role in cytotoxic activity, was not a marker within our healthy control group, thus showing selectivity of disease states on our chip (Osińska et al., 2014). A2 and B1 serum cohorts shared 3 analytes not found in other cohorts: HSP70, IL-1β, and IL-2. HSP70, a stress protein known to induce inflammation and has been linked to pathogenesis of Kawasaki disease (Lu et al., 1998; Hulina et al., 2018) was not present in A2 saliva but was present in B1 saliva samples. ACE2 (angiotensin converting enzyme-2) is a monocarboxypeptidase found within cell membranes or as a soluble protein throughout the body (Jiang et al., 2014). It is a member of the renin-angiotensin system (RAS) and has been implicated in diabatic cardiovascular complications and chronic heart failure; elevated expression has been linked to severe COVID-19, and it is a receptor for SARS-CoV2 entry into the cell (Patel et al., 2013; Jiang et al., 2014; Beyerstedt et al., 2021).We detected ACE2 in the serum biomarker signatures for A1, A2, and B1. While ACE2 levels in cohorts A1 and A2 are consistent with recent scientific literature, it is not a known biomarker in Kawasaki disease, despite the fact that cardiac disease (coronary artery aneurysms, myocarditis, pericarditis, congestive heart failure, pericardial effusion, and arrhythmias) is a complication in these patients (Tizard, 2005). The detection of ACE2 within cohort B1 could suggest the presence of cardiac disease within individuals of this group.

We have demonstrated the versatility of the GCFP microarray, the option to manipulate the composition of the microarray features, and the potential to detect over 1000 analytes in one assay (Guignon, and Lynes, 2014; Yuk et al., 2013; Molony et al., 2012; Unfricht et al., 2005; Marusov et al., 2012; Reilly et al., 2006). In the current study, using a small sample volume (70–80 µL) of either saliva or serum, we were able to begin to identify an initial biomarker signature for the different cohorts studied, showing that GCFP microarrays can be a potential novel approach for MIS-C disease diagnosis. ELISA or microsphere immunoassay (MIA), the gold standards for analyte measurement, use larger sample volume than GCFP, an especially important consideration with pediatric patients. We were also able to modify the protocol to increase assay sensitivity. MIA data in this study revealed four potential new GCFP targets, IL-10, IL-13, CXCL11 and SARS-COV-2 spike antigen, that can be added to the second-generation GCFP chip. By including more biomarkers on the chip, we can establish a more specific signature for each disease state tested, including indicators of individual organ function as well as systemic health. We are also able to detect disease state variation between patients within a disease group, which could potentially help with individualized diagnostics and treatment and provide faster treatment options per patient. It is important to note that negative markers (ones that do not appear as signals in these cohorts) are also informative and may be useful in excluding patients as belonging to one of these cohorts who present with those markers. We started building the first-generation GCFP chip at the beginning of the COVID pandemic. In future studies, ongoing research will be utilized to design a microarray in the second-generation chip that will produce biosignature that is even more specific and sensitive to MIS-C. Our data demonstrate GCFP microarrays as a novel, professional point of care diagnostic tool.

## The Connecticut Children’s COVID Collaborative

**Salazar, Juan C**, Connecticut Children’s Medical Center, Hartford, Connecticut 06106, United States, and University of Connecticut Health Center, Farmington, Connecticut 06030, United States; **Lynes, Michael A**, University of Connecticut, Storrs, Connecticut 06269, United States; **Lawrence David A,** Wadsworth Center, New York State Department of Health, Albany, New York 12208, United States, and University at Albany School of Public Health, Rensselaer, New York 12144, United States; **Brimacombe, Michael**, Connecticut Children’s Medical Center, Hartford, Connecticut 06106, United States, and University of Connecticut Health Center, Farmington, Connecticut 06030, United States; **Carroll, Christopher L,** onnecticut Children’s Medical Center, Hartford, Connecticut 06106, United States, and University of Connecticut Health Center, Farmington, Connecticut 06030, United States; **Carson, Kyle J**, Wadsworth Center, New York State Department of Health, Albany, New York 12208, United States; **Dagenais, Taylor RT**, Big Rose Web Design LLC, Middleton, Wisconsin 53562, United States; **De La Cruz Macías, Catalina**, Centro de Estudios en Infectología Pediátrica, Cali, Colombia; **El Chebib, Hassan**, Connecticut Children’s Medical Center, Hartford, Connecticut 06106, United States, and University of Connecticut Health Center, Farmington, Connecticut 06030, United States; **George, Joshy**, The Jackson Laboratory for Genomic Medicine, Farmington, Connecticut 06032, United States; **Ghassabian, Akhgar**, New York University Grossman School of Medicine, New York, New York 10016, United States; **Giles, Steven S**, Big Rose Web Design LLC, Middleton, Wisconsin 53562, United States; **Graf Jeorg**, University of Connecticut, Storrs, Connecticut 06269, United States; **Gunter, Courtney**, The Jackson Laboratory for Genomic Medicine, Farmington, Connecticut 06032, United States; **Herbst, Katherine W**, Connecticut Children’s Medical Center, Hartford, Connecticut 06106, United States; **Hawley, Kelly L**, Connecticut Children’s Medical Center, Hartford, Connecticut 06106, United States, and University of Connecticut Health Center, Farmington, Connecticut 06030, United States; **Hogan, Alexander H**, Connecticut Children’s Medical Center, Hartford, Connecticut 06106, United States, and University of Connecticut Health Center, Farmington, Connecticut 06030, United States; **Jadhav, Aishwarya**, Wadsworth Center, New York State Department of Health, Albany, New York 12208, United States; **Kozhaya, Lina**, The Jackson Laboratory for Genomic Medicine, Farmington, Connecticut 06032, United States; **Lee, William T**, Wadsworth Center, New York State Department of Health, Albany, New York 12208, United States, and University at Albany School of Public Health, Rensselaer, New York 12144, United States; **López, Eduardo L**, Centro de Estudios en Infectología Pediátrica, Cali, Colombia; **Maltz-Matyschsyk**, Michele, University of Connecticut, Storrs, Connecticut 06269, United States; **Melchiorre, Clare K**, University of Connecticut, Storrs, Connecticut 06269, United States; **O’Sullivan, Brandan**, University of Connecticut, Storrs, Connecticut 06269, United States; **Radolf, Justin D**, University of Connecticut Health Center, Farmington, Connecticut 06030, United States; **Unutmaz, Derya**, The Jackson Laboratory for Genomic Medicine, Farmington, Connecticut 06032, United States.

## Data Availability

The original contributions presented in the study are included in the article/Supplementary Material, further inquiries can be directed to the corresponding author.
