# Supplementary figures and images for: Development of a biomarker signature using grating-coupled fluorescence plasmonic microarray for diagnosis of MIS-C
Source: Front Bioeng Biotechnol. 2023 Mar 31;11:1066391. doi: 10.3389/fbioe.2023.1066391 (PMC10102909; doi:10.3389/fbioe.2023.1066391)

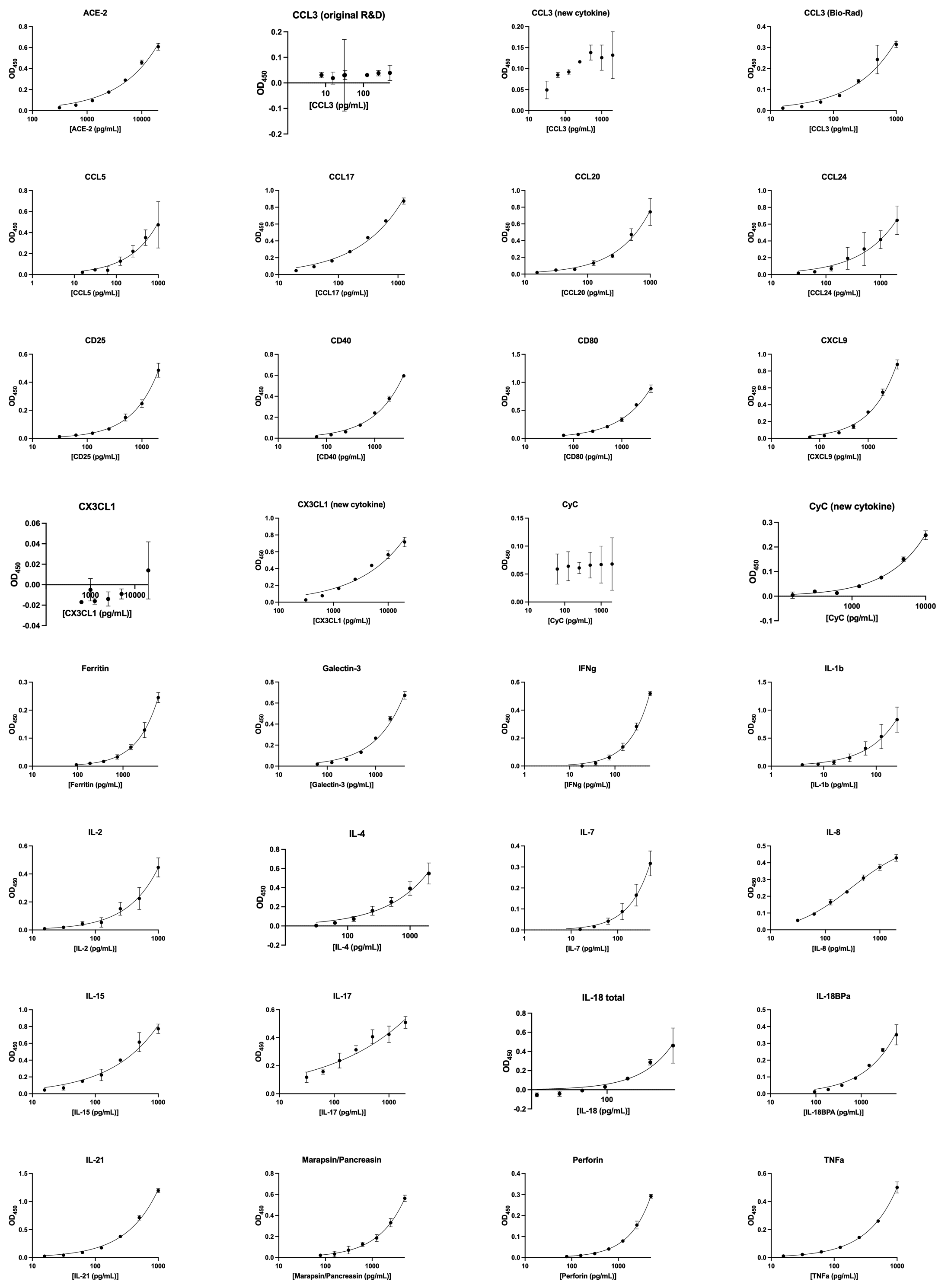

Supplement: Supplementary file 1 [file Image1.TIFF]
