# Supplementary material for: Development of a biomarker signature using grating-coupled fluorescence plasmonic microarray for diagnosis of MIS-C
Source: Front Bioeng Biotechnol. 2023 Mar 31;11:1066391. doi: 10.3389/fbioe.2023.1066391 (PMC10102909; doi:10.3389/fbioe.2023.1066391)
Supplement: Supplementary file 2 [file Table1.DOCX]

Supplementary Material:

Methods:

**Matched pair Enzyme-Linked Immunoassay (ELISA) reagent validation**

Antibody validation was performed by ELISA. Capture antibody (R&D Systems Inc, MN, USA; Mabtech AB, Nacka Strand, Sweden; CusaBio Technology LLC, TX, USA; Bio-Rad Laboratories, CA, USA) was diluted in PBS and 100μL /well was used to coat wells of an Immulon II HB flat bottom micrometer plate (Thermo Fisher Scientific, MA, USA). After incubation at room temperature overnight, the plate was aspirated and washed with PBST (160g NaCl, 4g KCl, 4g Kh_2_PO_4_, 23g NaHPO_4,_ 5mL Tween 20, 4g NaN_3_ dissolved into 20L H_2_0) using an ELx405 automated plate washer (BioTek), then blocked with 250μL of 2% BSA in PBS for 1.5h at RT. The plate was washed and incubated with 100μL of diluted recombinant cytokine in reagent diluent (1% bovine serum albumin in PBS) or PBS, according to kit instructions, for 2h at RT. Following this incubation, the plate was washed again and incubated with 100μL /well of manufacturer’s recommended concentration of secondary, biotinylated, antibody for 2h at RT. After washing, 100μL/well of recommended concentration of streptavidin-HRP was added to wells and incubated for 20 minutes at RT. After a final wash, 100μL/well of a 1:1 mixture of color reagent A (H_2_O_2_) and color reagent B (TMB (3,3’,5,5’-Tetramethylbenzidine) substrate was added to the plate and incubated for 20 minutes at RT in the dark. 50μL of 2N H_2_SO_4_ was added to stop the reaction and color production was measured at 570- 450nm in a Spectramax i3x plate reader (Molecular Devices, CA, USA).


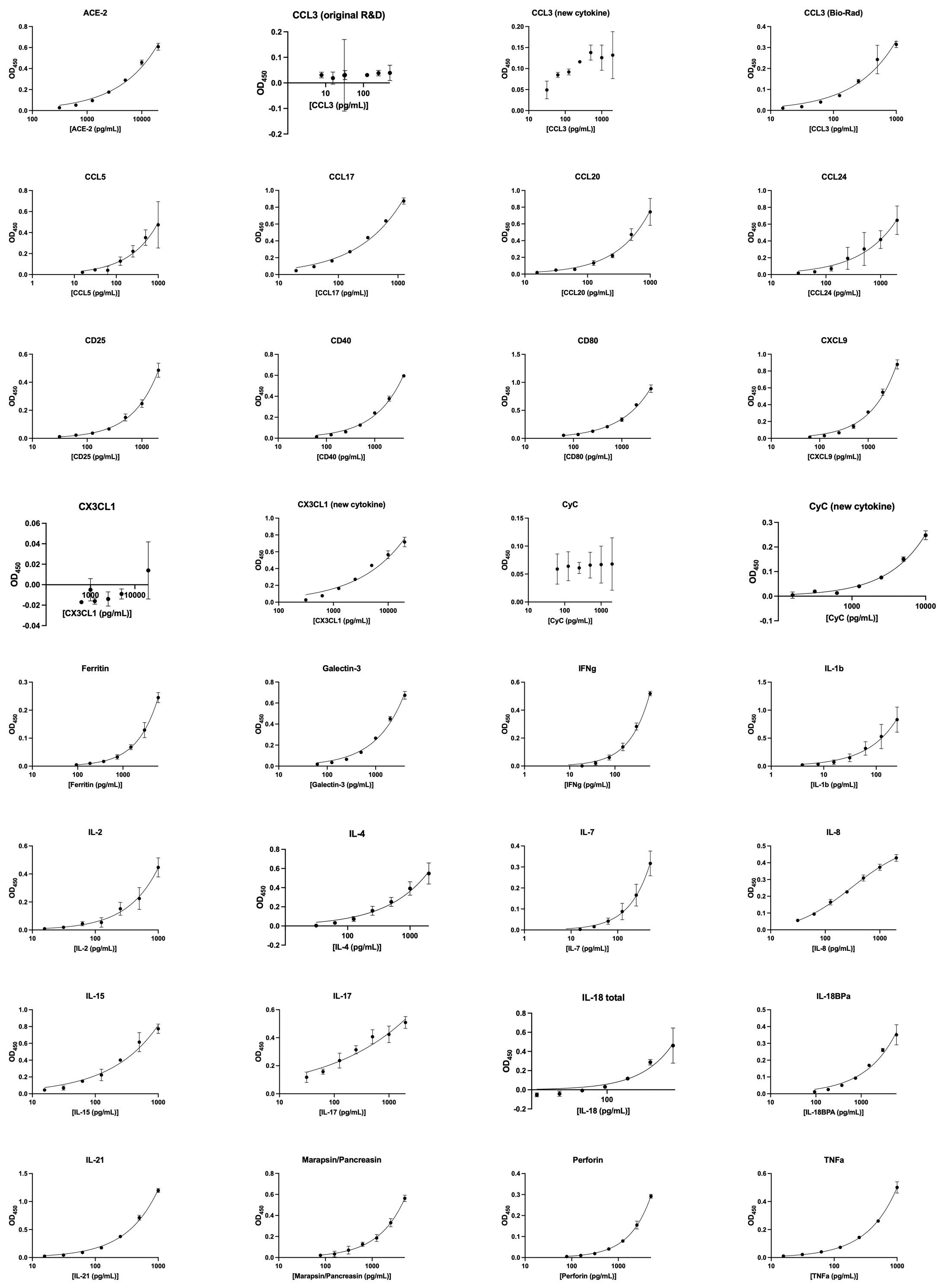


**FigS1.** **Matched pair Enzyme-Linked Immunoassay (ELISA) reagent validation results.** Forty-two commercially available matched pair antibodies were validated by ELISA using recombinant proteins. Nine commercially available kits that did not meet our ELISA standards when performed following manufacture protocols and were omitted from further analysis.

**Table S1** MIA (pg/mL) data for individual samples run over GCFP chip

| Cohort | Study ID | IFNγ | CCL20 | IL17A | IL1β | IL2 | IL21 | IL4 | IL6 | IL7 | | CCL3 | TNFα |
| --- | --- | --- | --- | --- | --- | --- | --- | --- | --- | --- | --- | --- | --- |
| A1 | 2100410146 | 17.99 | 22.46 | 25.39 | 2.64 | 3.09 | 11.67 | 15.17 | 12.17 | 8.63 | 15.05 | | 14.70 |
| A1 | 2100410150 | 9.73 | 19.91 | 11.26 | 1.74 | 1.37 | 4.41 | 19.56 | 21.61 | 9.23 | 9.61 | | 21.01 |
| A1 | 2100410042 | 41.67 | 29.65 | 12.21 | 2.64 | 5.26 | 7.64 | 113.49 | 22.78 | 23.18 | 19.02 | | 9.52 |
| A1 | 2100410049 | 24.95 | 26.19 | 6.26 | 1.12 | 2.17 | 3.09 | 32.05 | 4.76 | 18.65 | 30.83 | | 16.79 |
| A1 | 2100410142 | 17.99 | 34.28 | 10.61 | 1.77 | 1.42 | 3.30 | 8.15 | 7.88 | 5.08 | 40.25 | | 76.752 |
| A1 | 2100410242 | 25.75 | 81.5 | 4.4 | 1.387 | 2.145 | 2.513 | 5.59 | 7.747 | 6.06 | 89.34 | | 22.52 |
| A1 | 2100430005 | 160.96 | 42.86 | 16.28 | 4.48 | 6.17 | 9.59 | 21.96 | 1.85 | 11.10 | 20.86 | | 9.73 |
| A1 | 2100430012 | 98.53 | 29.89 | 13.55 | 2.92 | 4.22 | 6.64 | 40.44 | 0.47 | 10.75 | N/A | | 33.91 |
| A1 | 2100440004 | 73.92 | 85.147 | 11.612 | 2.03 | 3.94 | 6.7 | 10.84 | 1.02 | 14.17 | 22.32 | | 66.20 |
| A1 | 2100440006 | 102.4 | 54.86 | 16.93 | 2.16 | 3.241 | 5.99 | 9.48 | 7.56 | 11.60 | N/A | | 14.20 |
| A2 | 2100410124 | 18.11 | 26.4 | 16.94 | 10.07 | 9.83 | 20.0 | 1551.95 | 96.42 | 22.84 | 53.88 | | 14.73 |
| A2 | 2100410131 | 14.47 | 22.47 | 19.1 | 3.86 | 5.47 | 10.56 | 56.27 | 11.34 | 13.20 | 23.88 | | 24.94 |
| A2 | 2100410166 | 46.92 | 23.09 | 7.7 | 4.51 | 3.62 | 7.18 | 809.86 | 105 | 21.76 | 18.85 | | 18.59 |
| A2 | 2100410168 | 79.44 | 50.76 | 11.85 | 3.12 | 4.62 | 6.7 | 373.52 | 29.9 | 15.22 | 53.33 | | 77.09 |
| A2 | 2100410181 | 185.0 | 213.47 | 18.34 | 2.03 | 3.62 | 6.1 | N/A | 118 | 14.14 | 8.61 | | 43.39 |
| A2 | 2100410184 | 77.08 | 43.18 | 23.77 | 6.11 | 6.26 | 10.49 | 1851.39 | 157.8 | 17.92 | 80.81 | | 27.51 |
| A2 | 2100430007 | 176.8 | 30.19 | 11.26 | 4.85 | 6.73 | 6.87 | 38.37 | 2.30 | 28.32 | 17.55 | | 12.54 |
| A2 | 2100430011 | 240.3 | 45.68 | 30.54 | 8.76 | 10.63 | 14.15 | 727.89 | 70.83 | 16.46 | 52.53 | | 22.87 |
| A2 | 2100440014 | 233.7 | 51.14 | 52.66 | 6.49 | 7.06 | 20.04 | 605.91 | 58.72 | 27.53 | 43.91 | | 29.89 |
| A2 | 2100440026 | 44.56 | 54.32 | 15.37 | 3.99 | 7.13 | 4.84 | 35.42 | 2.91 | 15.39 | 13.60 | | 17.7 |
| A2 | 2100440038 | 53.7 | 42.57 | 14.93 | 3.34 | 6.06 | 8.82 | 101.33 | 36.29 | 16.34 | 47.10 | | 57.18 |
| A2 | 2100440051 | 44.87 | 47.10 | 21.02 | 4.07 | 7.49 | 7.80 | 95.68 | N/A | 17.75 | 22.35 | | 18.62 |
| B1 | 2100410153 | 28.27 | 29.54 | 34.51 | 7.47 | 8.77 | 18.13 | 119.17 | 22.7 | 14.95 | 29.63 | | 38.45 |
| B1 | 2100410009 | 35.19 | 18.97 | 8.14 | 2.11 | 2.87 | 8.19 | 500.77 | 87.09 | 11.8 | 41.52 | | 9.81 |
| B1 | 2100410114 | 22.69 | 40.46 | 26.17 | 8.92 | 8.39 | 10.67 | 528.42 | 95.82 | 29.19 | 47.82 | | 27.94 |
| B1 | 2100410162 | 70.42 | 43.18 | 14.61 | 5.67 | 5.57 | 7.93 | 1414.72 | 162.4 | 13.27 | 63.13 | | 6.66 |
| B1 | 2100410195 | 97.37 | 47.37 | 34.84 | 3.83 | 4.99 | 5.89 | 79.25 | 42.34 | 14.09 | 20.29 | | 15.02 |
| B1 | 2100410208 | 95.32 | 73.54 | 20.01 | 6.12 | 7.85 | 13.23 | 786.04 | 116.8 | 15.47 | 61.71 | | 70.39 |
| B1 | 2100410220 | 67.37 | 37.69 | 14.90 | 6.21 | 6.84 | 9.03 | 920.48 | 105.7 | 18.9 | 50.73 | | 14.47 |
| B1 | 2100430009 | 82.88 | 44.79 | 19.08 | 2.81 | 3.49 | 5.24 | 39.07 | 8.09 | 13.86 | 59.33 | | 85.50 |
| B1 | 2100440008 | 147.7 | 54.73 | 23.17 | 4.79 | 7.38 | 18.49 | 24.78 | 5.28 | 12.41 | 12.75 | | 9.96 |
| B1 | 2100440019 | 93.39 | 63.59 | 19.38 | 4.48 | 4.48 | 8.32 | 267.71 | 22.62 | 55.53 | 33.94 | | 57.48 |
| B1 | 2100440035 | 47.24 | 51.83 | 17.97 | 3.69 | 7.54 | 7.58 | 76.84 | 14.06 | 20.91 | 19.59 | | 14.47 |
| B1 | 2100440062 | 109.1 | 136.29 | 48.41 | 9.28 | 16.41 | 30.35 | 143.98 | 15.34 | 83.31 | 27.95 | | 18.94 |
| B2 | 2100410154 | 22.02 | 24.15 | 24.72 | 5.57 | 4.08 | 15.11 | 74.51 | 9.77 | 19.23 | 18.07 | | 28.34 |
| B2 | 2100410094 | 20.91 | 17.06 | 3.79 | 1.19 | 2.39 | 2.87 | 21.83 | 6.31 | 10.23 | < 0.3 | | 7.19 |
| B2 | 2100410099 | 49.65 | 29.98 | 10.85 | 2.89 | 3.56 | 12.36 | 724.66 | 141.3 | 15.74 | 42.33 | | 20.87 |
| B2 | 2100410018 | 47.01 | 27.14 | 16.90 | 5.28 | 7.56 | 9.85 | 235.52 | 56.30 | 25.04 | 38.24 | | 15.94 |
| B2 | 2100410128 | 16.18 | 22.58 | 19.70 | 3.86 | 4.29 | 9.51 | 319.57 | 37.43 | 11.81 | 20.90 | | 8.05 |
| B2 | 2100410130 | 11.1 | 34.29 | 9.37 | 1.94 | 1.61 | 2.97 | 18.72 | 2.56 | 6.44 | 15.01 | | 45.42 |
| B2 | 2100410136 | 26.26 | 45.1 | 18.86 | 4.86 | 6.4 | 11.25 | 80.88 | 12.04 | 16.33 | 16.60 | | 105.1 |
| B2 | 2100410139 | 11.1 | 17 | 27.98 | 1.81 | 1.62 | 6.14 | 54.29 | 2.25 | 12.06 | 12.73 | | 12.08 |
| B2 | 2100410165 | 85.13 | 37.52 | 25.05 | 4.11 | 10.11 | 6.49 | 169.61 | 26.54 | 12 | 19.99 | | 13.89 |
| B2 | 2100410223 | 54.96 | 45.73 | 11.99 | 3.78 | 5.67 | 4.53 | 14.75 | 0.79 | 15.68 | 42.47 | | 11.18 |
| B2 | 2100440013 | N/A | 62.42 | < 0.73 | <0.49 | < 0.49 | <0.24 | < 1.83 | 0.65 | 3.65 | 44.67 | | 98.61 |
| B3 | 2100410044 | 847.5 | 177.3 | 344.7 | 63.32 | 104.8 | 260.3 | 1775.5 | 227.8 | 155.9 | 147.6 | | 84.09 |
| B3 | 2100410047 | 76.16 | 32.09 | 22.40 | 2.24 | 5.95 | 6.38 | 22.93 | 7.53 | 15.58 | 20.62 | | 10.98 |
| B3 | 2100410178 | 177.4 | 48.64 | 54.23 | 4.73 | 13.26 | 12.92 | 30.39 | 3.34 | 13.31 | 31.18 | | 13.25 |
| B3 | 2100410179 | 125.9 | 35.24 | 38.67 | 6.02 | 7.25 | 10.76 | 815.69 | 83.03 | 17.26 | 39.91 | | 5.62 |
| B3 | 2100410180 | 53.19 | 24.63 | 8.84 | 2.57 | 3.98 | 4.71 | 49.15 | 2.77 | 8.42 | 18.47 | | 22.29 |
| B3 | 2100410186 | 124.6 | 56.26 | 49.42 | 46.75 | 37.12 | 6.28 | 220.27 | 3 | 11.76 | 12.93 | | 23.91 |
| B3 | 2100410224 | 106.0 | 28.51 | 26.22 | 7.34 | 12.14 | 12.46 | 52.22 | 7.09 | 20.61 | 23.51 | | 9.05 |
